# Differential gene expression in bovine endometrial epithelial cells after challenge with LPS; specific implications for genes involved in embryo maternal interactions

**DOI:** 10.1371/journal.pone.0222081

**Published:** 2019-09-05

**Authors:** Yongzhi Guo, Tom van Schaik, Naveed Jhamat, Adnan Niazi, Metasu Chanrot, Gilles Charpigny, Jean Francois Valarcher, Erik Bongcam-Rudloff, Göran Andersson, Patrice Humblot

**Affiliations:** 1 Swedish University of Agricultural Sciences, Department of Clinical Sciences, Division of Reproduction, Uppsala, Sweden; 2 Swedish University of Agricultural Sciences, Department of Animal Breeding and Genetics, Section of Molecular Genetics, Uppsala, Sweden; 3 Department of Gene Regulation, Netherlands Cancer Institute, Amsterdam, The Netherlands; 4 Department of Information Technology, University of the Punjab, Lahore, Pakistan; 5 Swedish University of Agricultural Sciences, Department of Animal Breeding and Genetics, Section of Bioinformatics and SLU Bioinformatics Infrastructure, Uppsala, Sweden; 6 Science for Life Laboratory, Uppsala University, Uppsala, Sweden; 7 Faculty of Veterinary Science, Rajamangala University of Technology Srivijaya, Nakorn sri thammarat, Thailand; 8 INRA, UMR1198 Biologie du Développement et Reproduction, Jouy-en-Josas, France; 9 Swedish University of Agricultural Sciences, Department of Clinical Sciences, Division of Ruminant Medicine, Uppsala, Sweden; University of Florida, UNITED STATES

## Abstract

Lipopolysaccharide (LPS) expressed on the surface of Gram-negative bacteria activates pro-inflammatory pathways, dys-regulates the function of endometrial cells and is a key player in the mechanisms involved in endometritis. This study aimed to investigate the effects of LPS on bovine endometrial epithelial cells (bEEC) from whole transcriptome with a special focus on genes involved in embryo-maternal interactions. Following *in vitro* culture, bEEC from three cows were exposed to 0, 2, and 8 μg/mL LPS for 24h. RNA samples extracted at 0 and 24 hours were analyzed by RNA sequencing (RNA-seq). At 24h, 2035 differentially expressed genes (DEGs) were identified between controls and samples treated with 2 μg/mL LPS. Gene ontology analysis showed that over-expressed DEGs were associated to immune response, response to stress and external stimuli, catalytic activity, and cell cycle. Genes associated with cell membrane and cell adhesion pathways were under-expressed. LPS induced changes in expression of specific genes related to embryo-maternal interactions including under-expression of eight members of the cadherin superfamily, over-expression of six members of the mucin family, and differential expression of a large set of genes binding the above molecules and of more than 20 transcripts coding for cytokines and their receptors. Type I interferon-τ dependent genes were also over-expressed. From a sub-set of 19 genes, (biological replicates of bEEC from cows taken at time 6 (n = 3), 24 (n = 6) and 48 hours (n = 3), and 2 technical replicates per sample) differential gene expression was confirmed by RT^2^-qPCR (r^2^ between fold changes at 24 hours by RT^2^-qPCR and RNA-seq = 0.97). These results indicate that LPS affects the function of bEEC in many ways by differential transcription, glycolytic metabolism and oxidative stress. Many transcriptomic signatures related to implantation and embryo maternal interactions were strongly affected by LPS. These results pave the way for further studies to investigate the duration of these changes and their possible impact on endometrial function and fertility.

## Introduction

Escherichia coli (*E*. *coli*) is commonly associated with uterine infection in post-partum cows [1, 2]. Lipopolysaccharide (LPS), at the outer membrane of *E*. *coli* and other Gram-negative bacteria, is one of the most powerful bacterial virulence factors triggering inflammation of the endometrium [3, 4]. Moreover, LPS has been reported to induce implantation failure, and pregnancy losses in the human species [5, 6]. LPS binding to toll-like receptors trigger pathways activating the production of pro-inflammatory cytokines [7, 8]. In addition, in different cell models including epithelial cells from different species, oxidative stress and glycolysis associated also to increased proliferation have been reported [9]. Cytokines including growth factors and their corresponding receptors expressed on endometrial cells play an important role in the processes regulating embryonic development, and in the interactions between the embryo and maternal cells, especially at time of implantation [10]. Successful implantation is the result of a very precise balance between cytokines which are necessary to the induction of uterine remodeling (such as LIF, IL-6, IL-17) and cytokines such as TGFβ, IL-10, IL-27, G-CSF limiting the inflammatory processes and driving the response of the maternal endometrium to embryo towards immune-tolerance [11]. In human and mice, it has been shown that other molecules such as Galectin-1 (Gal-1) are involved in the regulation of the differentiation of naïve CD4^+^ T cells to CD4^+^CD25^+^ regulatory T (Treg) cells, which are key effectors in orientating the innate immune response mechanisms towards immune-tolerance [12–14].

In ruminants, due to essential for maternal recognition of pregnancy and maintenance, a special attention has been paid to IFN-τ [15, 16]. Transcriptomic and functional studies in both the ewe and cow have shown that a large set of genes are over-expressed in the endometrium in response to IFN-τ [17]. Some of these genes such as *MX1* and *MX2* encode proteins that are also involved in the response to pathogens or disease [18].

Deviations in the elevation of pro-inflammatory cytokines in the uterine environment during the preimplantation period may damage endometrial cells, perturb embryo maternal interactions, and trigger DNA damage in embryonic cells. All these processes are potentially involved in implantation failures [5, 19].

More recently, with similar tools, the *in vitro* effects of LPS treatment on gene expression patterns were studied in primary cultures of mixed bovine epithelial and stromal endometrial cells, and gene expression of *SAMD9*, *PLAC8 and LGALS9* which encode proteins that are important for early pregnancy were over-expressed [20, 21].

In this study, we used RNA-sequencing technology to analyze with an unbiased approach, gene expression changes associated to the response of a pure population of bovine endometrial epithelial cells to a single challenge with *E coli* LPS. The data obtained here provide additional results on gene expression changes as a complement to former published information on the phenotypic response in terms of proliferation and survival of cells [9] and on the changes in proteomic profiles induced by LPS [22] in the same cell model. The present results show a wide range of responses of these cells to LPS, confirming the high pro-inflammatory role of this molecule and former information obtained on the corresponding pathways in endometrial cells and other types of epithelial cells [20, 23]. In addition, we provide novel information regarding the impact of LPS on sets of specific genes related to embyromaternal interactions and corresponding pathways which were here specifically scrutinized. Although incomplete due to the nature of this model based on a single cell type, these results contribute to understand the potential role of LPS on persistence of inflammation in the endometrium and its possible unfavorable effects on endometrial function possibly impairing fertility.

## Materials and methods

### Isolation of bovine endometrial epithelial cells (EEC)

The uterine horns from three Swedish Red Breed (SRB) cows were collected from slaughterhouse (Lövsta SLU, Uppsala, Sweden). Endometrial epithelial cells were separated then cultured according to procedures previously described [9]. Within one hour after slaughter, the endometrium was dissected into 5–6 cm long and 4–5 mm depth pieces. Tissue pieces were incubated for two hours at 39°C with collagenase IV (C5138, Sigma, Saint Louis, MO, USA) and hyaluronidase (250 U/mL) (H3506, Sigma) in PBS containing 2% BSA (Sigma). The suspension was then filtered through 250 μm gauze to remove mucus and undigested tissue. The filtrate was then passed through a 40 μm nylon sieve, which allowed the fibroblasts and blood cells to pass through while epithelial cells were retained. Epithelial cells were collected from the filter by backwashing with 30 mL PBS. Cells were centrifuged at 170 ×g for six minutes and the pellet re-suspended in three mL of PBS. Pellets were dispersed into a single cell suspension by passing through a fine gauge needle (20G). Cells were then cultured in F-12 medium (Dulbecco's modified eagle's medium, D6434, Sigma) containing 10% Fetal Bovine Serum (FBS), 1% Penicillin/streptomycin (5000 units/mL penicillin/streptomycin, Gibco, Carlsbad, CA, USA), 2 mM L-glutamine, 0.5% Liquid Media Supplement (ITS), and gentamycin (5 μg/mL) and nystatin (100 U/mL). Cells were seeded into a 25 cm^2^ ventilation flask. Cell cultures were kept in a water-jacked incubator with 5% CO_2_ at 39°C. Medium was changed every 1–2 days. Sub-cultivations were performed when epithelial cells attained 80 to 90% confluence [24, 25].

**Fig 1:**
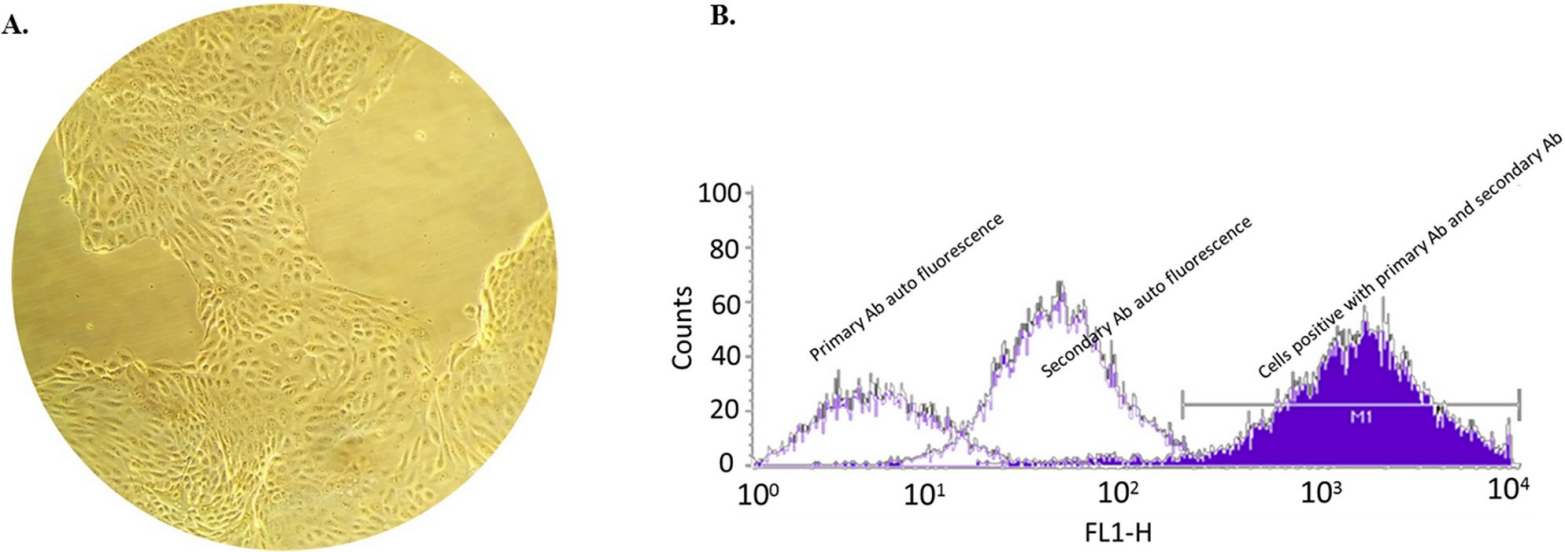
Morphology and purity of bovine endometrial epithelial cells. A. Cell morphology of bovine endometrial epithelial cells (bEECs) B. Representative Flow cytometric histogram of bEECs expressing the epithelial-specific cytokeratin-18 showing their appearance to the epithelial cell population. (98% of cells are in M1 region corresponding to this population).

The purity of bovine endometrial epithelial cell (bEEC) culture was estimated by morphological observation following three to five passages. Potential residual contamination of fibroblasts was eliminated between each passage by detaching with 3 mL tryp^LETM^ express (Gibco 12605) for two min at 39°C, and aspiration of the supernatant. Cells retained in the bottom were then viewed under the microscope to determine the presence of fibroblasts [26]. After passage 3 and before LPS challenge, no fibroblasts were found by microscopic observation (Fig 1A). The purity of the epithelial cell culture was checked by flow-cytometry labelling cytokeratin (Primary Anti-cytokeratin 18 Ab, Abcam, Cambridge, UK, cat ab 668, and secondary Anti-alex 488 Ab, cat ab175473 used following manufacturer’s instructions). From passage 2 and thereafter, more than 98% of cells expressed cytokeratin, confirming the very high purity of the cell culture system (Fig 1B).

### LPS challenge and Isolation of total RNA

As previously described [22], bEEC were challenged on passage 5, with 0, 2, and 8 μg/mL LPS from *E*.*coli* (O111:B4; Sigma). These concentrations of LPS reflected concentrations found in uterine fluid in cases of clinical endometritis [27, 28]. Moreover, we did choose to perform this study with these LPS dosages and a pure population of endometrial epithelial cells as used in our former experiments, in cell survival, proliferation, cytokine production and proteomic profiles 72 hours post LPS exposure [22]. At the time of challenge and 24 hours later, cells were detached by 5 mL trypLE^TM^ express (Gibco 12605) and washed with DPBS (Gibco). The present study in changes in gene expression by RNAseq and in subsequent validation studies were performed at earlier stage when compared to cell survival and proteomics, which corresponded also well with the design of previous studies showing changes in expression of TLR4 mRNA in bovine endometrial cells [29, 30]. Samples of two million cells were frozen at -80°C. Total RNA was extracted with the Allprep DNA/RNA/miRNA Universal Kit (Qiagen, Darmstadt, Germany, Cat. No. 80224), and the respective concentrations of total RNA and mRNA (RIN) was determined from Bioanalyzer 2100 (Agilent Technologies, Santa Clara, CA). RNA samples had 28S/18S ratios ranging from 1.8 to 2.0, concentrations from 805–1580 ng/μl, and RIN values of RNA ranged from 8.8 to 10.0 (S1 Table).

### Library preparation and sequencing

**Fig 2:**
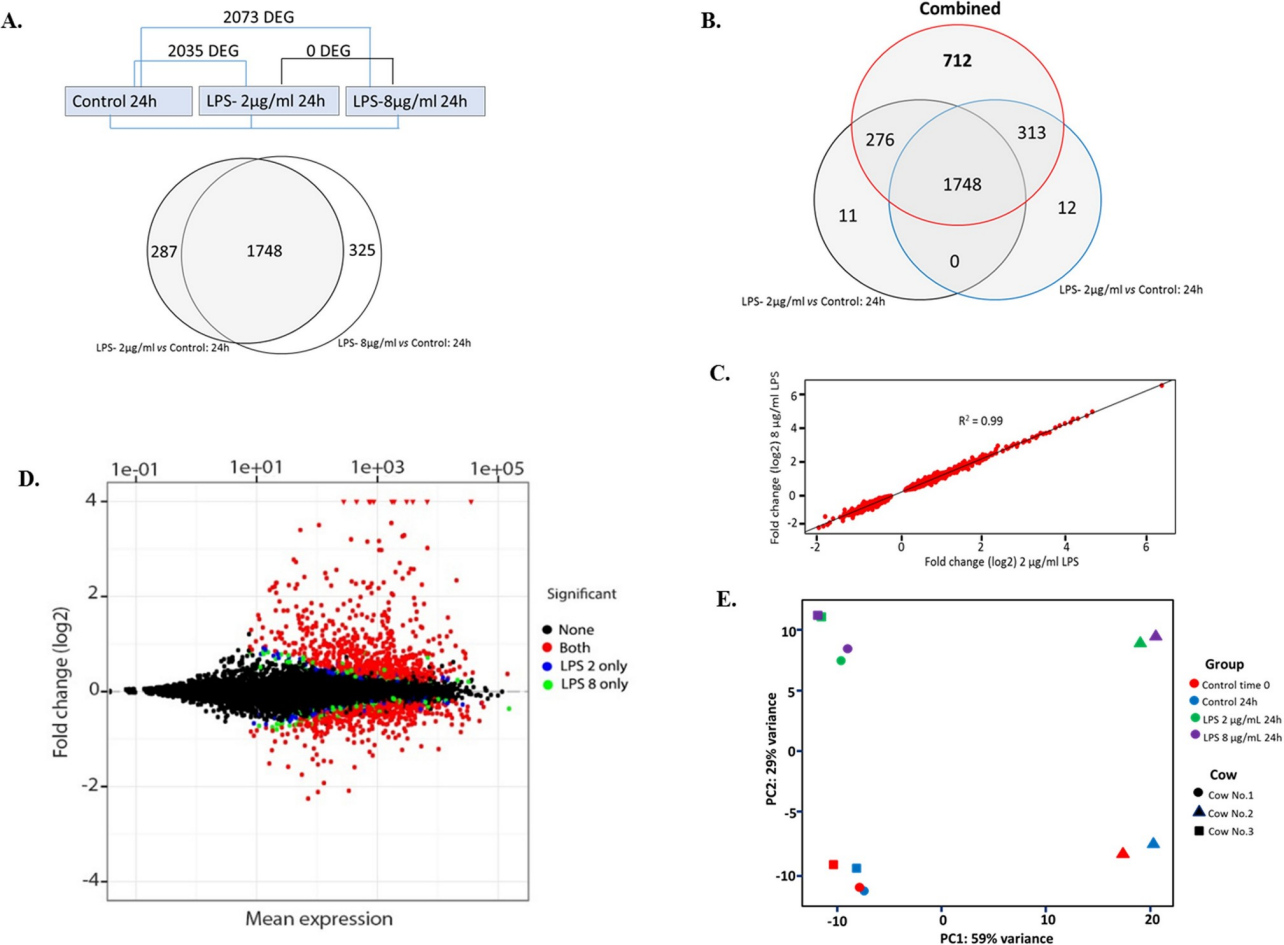
Comparison of differentially expressed genes. A. Design of comparisons made between groups (shown above) and Venn diagram of differentially expressed genes (DEGs) between each LPS-treated groups and controls (below). B. Venn diagram including the distribution of DEGs found in both LPS treatments together (combined 2 and 8 μg/mL LPS) versus control cells showing that most of the genes which were not common in A expressed similar changes as becoming DEGs in the “combined” comparison. C. Correlation of log2 fold changes between the 2 and 8 μg/ml LPS-treatment. D. MA-plot of all DEGs identified in the different LPS treatments, log2 fold changes for each gene between two samples (on the y-axis) versus the mean expression signal (on the x-axis). Blue (2μg/ml LPS), green (8 μg/ml LPS) and red dots (common) represent the genes with significant differential expression (Adjusted P value <0.05 from Benjamini Hochberg correction). Significant mean positive log2 fold changes corresponds to increased expression, whereas negative values corresponds to decreased expression. E. Principal Component Analysis (PCA) from the top 500 most variably expressed genes at 24h post LPS. Symbols for each cow have a different shape and with a different color for each condition as indicated to the right. Axis 1 reflects individual variation in basic gene expression between individuals whereas axis 2 reflects the effect of LPS treatment.

Sequencing libraries from 12 samples (for each cow, on Time 0, and with 0, 2, and 8 μg/mL LPS at 24h, Fig 2) were prepared by the sequencing platform (Science for Life Laboratory, Uppsala University; https://www.scilifelab.se/) from 1μg of total RNA using the TruSeq stranded mRNA sample prep kit including polyA selection (RS-122-2101/2, Illumina Inc, San Diego, CA, USA) (15031047, revE). RNA sequencing was then performed using the Illumina HiSeq2500 system. Paired-end 125bp reads were obtained with sequence depth of about 30 million reads per sample.

### Data quality control and read abundance calculation

The quality of the RNA-Seq data generated from control (control time 0, n = 3 and control 24h, n = 3) and LPS-treated samples (2 μg/mL, n = 3 and 8 μg/mL, n = 3) were first studied using FastQC 0.11.2 [31]. The adapter sequences and low quality reads were then filtered with Trimmomatic 0.32 [32], and reads were mapped to the *Bos Taurus* reference genome bosTau6 with STAR 2.4.0 software [33]. Of the filtered reads, approximately 97% were mapped to the *Bos taurus* reference genome. Uniquely mapped reads were counted to ENSEMBLv78 annotations with HTSeq 0.6.1 for downstream differential expression analyses [34]. The RNA-Seq experiment generated an average of 26.35 ± 1.96 (mean ± SD) million uniquely mapped reads per sample (S1 Table).

### Principal component analysis (PCA) and identification of significantly differentially expressed genes (DEGs) and pathway analysis

PCA and the identification of DEGs were performed with DESeq2 version 1.6.3 [35]. For the PCA, raw counts were normalized and log transformed with rlog function provided in DESeq2. After differential expression analysis, the resulting p-values were adjusted for multiple testing using the Benjamini–Hochberg procedure. DEGs with adjusted *p*-values < 0.05 were regarded as significant. DEGs were identified from analysis of paired wise comparisons (each cow individual untreated samples from which cell culture was issued taken as its own control) *i*) between control at time 0 and control at 24h, *ii*) between control at 24h *vs* 2 μg/mL and 8 μg/mL LPS-treated at 24h (LPS dosages taken individually), *iii*) between 2 μg/mL and 8 μg/mL LPS-treated samples at 24h, *iv*) between control samples and LPS-treated samples at 24h (2 and 8 μg/mL LPS taken together), (Fig 2A). Gene ontology (GO) and Kyoto Encyclopedia of Genes and Genomes (KEGG) pathway enrichment were determined using DAVID 6.7 [36, 37] with all Ensembl genes as background [38]. For GO term analysis, the DEGs were analyzed by running queries for each DEG against GO database, which provides information related to three ontologies: biological process (BP) cellular component (CC), molecular function (MF).

MA-plots are used to visualize the differential gene analysis results (a scatter plot of log_2_ fold changes for each gene between two samples (on the y-axis) versus the mean expression signal (on the x-axis)). Consequently, genes with similar expression levels in two samples appear around the horizontal line y = 0. Analysis of genes possibly involved in disease and physiological system functions was based on GeneCards database (http://www.genecards.org/) which integrates gene-centric data from around 125 web sources.

### RT^2^-qPCR validation and changes in gene expression with time

Validations of relative gene expression levels obtained from RNA-seq were performed by RT^2^-qPCR (RT^2^ Profiler^TM^ PCR Array: CAPB13612, QiaGen, Hilden, Germany), using a subset of samples from those used for the RNA sequencing (Control 24 hours and 24 hours after 2 μg/mL LPS) and another set of samples issued from the culture of cells from 3 additional cows. Cells from these cows were exposed to 2 μg/mL LPS for 6, 24 and 48 hours and comparisons made with controls obtained at the same time. Two technical replicates were done on each biological replicate of bEEC from cows taken at time 6 (n = 3), 24 (n = 6) and 48 hours (n = 3) following LPS exposure. Nineteen different genes (*IFIT3*, *MUC1*, *IFIT1 MUC13*, *ITGB3*, *ITGB6*, *CDH26*, *PKP1*, *STAT1*, *NFKB1*, *SMAD3*, *LIF*, *MMP1*, *MMP7*, *MMP13*, *TGFB2*, *LGALS1*, *LGALS3*, *and LGALS9)* were selected for validation based on their biological relevance and on their significance to cover the full range of differential expression observed from RNAseq. When several genes from the same family were differentially expressed we took one member from list from highly significant ones and one member for which the adjusted value was close to 0.05. Results were normalized from changes for three housekeeping genes used as reference (*TBP*, *ACTB* and *GAPDH*). As differences were expressed the same way with the 3 reference genes, results are presented while using *ACTB* as reference.

### Statistical analyses

Generalized linear model was fitted and Wald test was performed to determine which of the observed fold changes were significantly different between pairwise analyses of controls and treatment groups. The ANOVA-like DESeq2 likelihood ratio statistic (LRT) test was used to test multiple levels of control and treatment at once. Adjusted p values *p* < 0.05 were considered to determine DEGs.

General linear models were used (proc GLM, SAS Ver 9.4) to analyze RT^2^qPCR data. Cow and time (6, 24, 48 hours) were included as main effects in the models potentially influencing the differences in expression between control vs 2 μg/mL LPS (y = expression LPS time x–expression Control time x) and ratio of expression; z = (expression LPS time x–expression Control time x) / expression Control time x). In this case, the paired wise statistics compares the observed value “y” or “z” to “0” to be observed in case of the “null hypothesis” (lack of effect). As the effects were systematically the same for the 2 above variables only “z” results are presented. When necessary log transformation was used to normalize the expression data for some genes. For the sake of clarity, non-transformed ratios are presented. The significance of ndividual comparisons between times were subsequently analyzed by using multiple comparison adjustments (“Scheffé” test and contrast option under GLM).

The correlation between fold changes obtained from RNA-seq and those from the RT^2^-qPCR experiment for the 19 genes, (mean fold change from 3 cows at 24 hours following 2 μg/mL LPS from the two techniques) was studied by using Spearman correlation coefficient.

## Results

### Overall differential gene expression analysis

The number of differentially expressed genes (DEGs) between control and LPS groups at 24 hours post-challenge are presented in Fig 2A. Overall, 2035 and 2073 DEGs were identified between control cells and cells treated with 2 and 8 μg/mL LPS, respectively. No gene was identified as being differentially expressed between the two different LPS dosages employed, allowing the two LPS-treated samples from each cow to be considered as biological replicates in some of the analyses. A total of 1748 DEGs were found to be common between the two treatment groups. Nearly 700 additional DEGs were identified when comparing both LPS treatments taken together (groups of 2 and 8 μg/mL LPS) to controls indicating that in almost all cases, gene expression levels for these genes show similar variation in response to LPS for both treatment groups (Fig 2B). This is further confirmed by the correlation coefficient between fold changes observed with 2 and 8 μg/mL LPS is r^2^ = 0.99 (Fig 2C).

A slightly higher number of genes were identified as being over-expressed than under-expressed. From the 2035 genes significantly de-regulated 24h after exposure to 2μg/ml *E*.*coli* LPS, 1066 were over-expressed (52.4%), and 969 under-expressed (47.6%). Furthermore, higher fold changes were in general observed for the over-expressed genes when compared to genes with reduced expression levels (Fig 2D). DEGs, which were found as significant only for one of the LPS dosages (for the 2 and 8 μg/mL LPS, respectively) were close to the significance cut off level (*p* < 0.05). Due to the large common number of DEGs found and to the high degree of similarity between transcriptomic profiles observed between the two dosages (*r*^2^ = 0.99; Fig 2D), the detailed analysis and interpretation was based mainly on the comparison between control *vs* 2 μg/mL LPS at 24h. The common response of LPS groups when compared to controls was further revealed from the principal component analysis (PCA) made from the top 500 genes displaying highest variability across samples (Fig 2E). Despite that bEECs obtained from one cow displayed an atypical basic expression pattern in controls, the PCA shows that response to LPS treatment appeared very consistent with the differential gene expression observed for the two other cows.

### Gene ontology analysis of the DEGs

**Fig 3:**
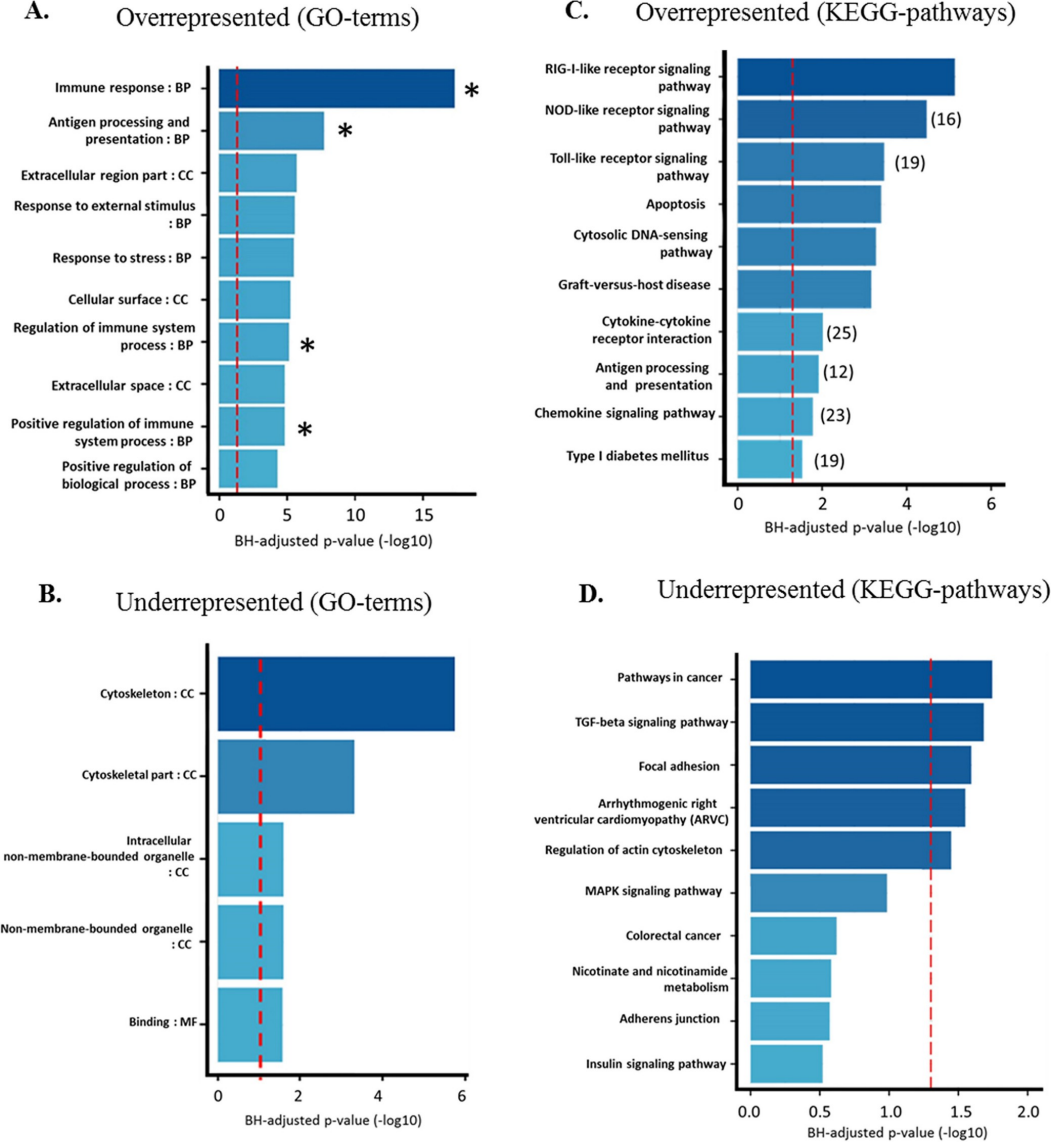
Gene ontology (GO) and KEGG pathways analysis of over-expressed and under-expressed DEGs. A. Overrepresented GO-terms pathways, * = pathways related to immune function. B. Underrepresented GO-terms pathways C. Overrepresented KEGG pathways D. Underrepresented KEGG pathways. Vertical red dotted bars indicate the cut-off level for significance (P < 0.05). P-values were adjusted by Benjamini-Hochberg correction for multiple testing.

DAVID 6.7 was used to identify biological pathways, through associated over-represented biological GO-terms corresponding to significantly DEGs in cells exposed to LPS. GO enrichments of the DEGs were categorized into 484 functional groups with a corresponding adjusted *p* value < 0.001. This total included 274 over-expressed and 210 under-expressed groups. For the over-expressed groups, 214 (78.1%), 15 (5.5%) and 45 (16.4%) were categorized into biological process (BP) cellular component (CC), molecular function (MF), respectively. For the under-expressed groups, 134 (64%), 52 (25%), and 24 (11%), were categorized into BP, CC, and MF, respectively (S2 Table). The dysregulated pathways with the most significant adjusted *p* values are illustrated in Fig 3. Gene ontology analysis showed that four out of the top 10 significantly overrepresented pathways were related to innate immune response, inflammatory response and antigen processing and presentation (Fig 3A, S1 Fig and S3 Table). Response to stress and external stimuli, transcription, catalytic activity and glycolysis were also highly overrepresented. The top five underrepresented GO-terms are related to cell structures such cytoskeleton, cell membrane, binding and organelle (Fig 3B and S4 Table).

### KEGG pathway analysis of the DEGs

Overall, the DEGs were significantly enriched in 22 different KEGG pathways (adjusted *P*-values < 0.05) including 11 of overrepresented pathways and 11 of underrepresented pathways (S5 and S6 Tables). Similarly to the above, six out of 10 overrepresented pathways were related to inflammation (Nucleotide-binding, oligomerization domain (NOD)-like receptor signaling pathway with 16 DEGs enriched, Toll-like receptor signaling pathway (19 DEGs), cytokine-cytokine receptor interaction (25 DEGs), antigen processing and presentation (12 DEGs), chemokine signaling pathway (23 DEGs), and apoptosis (19 DEGs) (Fig 3C). Three out of 10 of the underrepresented pathways were related to focal adhesion, regulation of actin cytoskeleton and adherent junction, which are protein complexes that are present at cell–cell junctions in epithelial and endothelial tissues (Fig 3D).

**Fig 4:**
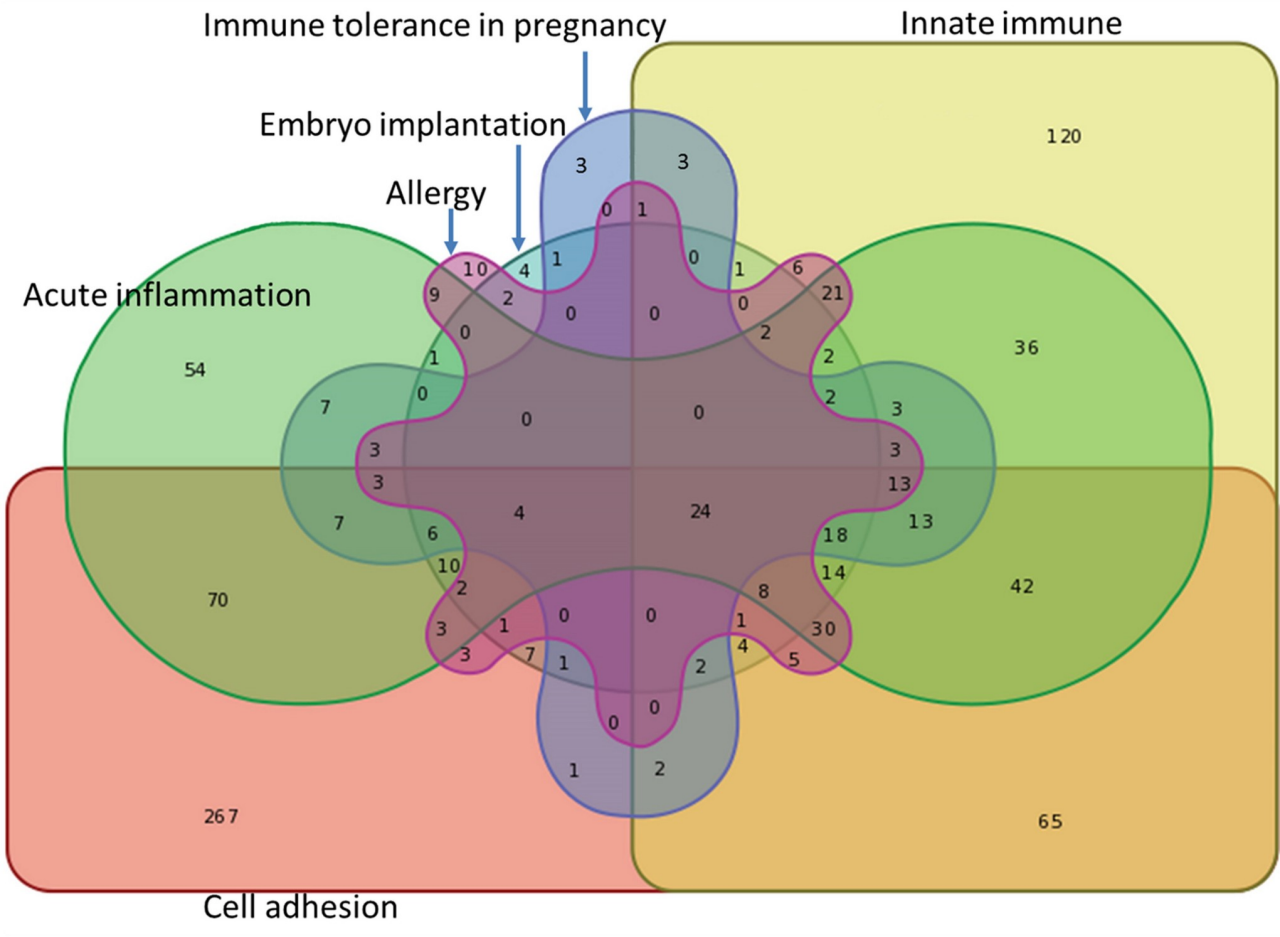
Comparison of DEGs after challenge with LPS to GeneCards database. Numbers of DEGs participating in acute inflammation (410 DEGs), innate immune (441 DEGs), immune tolerance in pregnancy (120 DEGs), allergy (153 DEGs), cell adhesion (626 DEGs) and embryo implantation (118 DEGs). The figure illustrates the relatively high number of DEGs common to two pathways and 24 common DEGs were involved in all above mentioned biological processes (http://www.genecards.org/).

Comparing results from the present RNA-seq data to GeneCards database (*http*:*//www*.*genecards*.*org*), we found different numbers of DEGs participating in acute inflammation (410 DEGs), innate immunity (441 DEGs), immune tolerance in pregnancy (120 DEGs), allergy (153 DEGs), and cell adhesion (626 DEGs). As embryo implantation is associated to inflammatory processes we found here also a quite high number of genes corresponding to this process through this classification (118 DEGs) (Fig 4 and S7 Table). Twenty four common DEGs (*EDN1*, *TGFB2*, *TGFB3*, *FAS*, *C3*, *ICAM1*, *CXCL8*, *IL1RN*, *TP53*, *TNF*, *IL1A*, *CAT*, *F5*, *NFKB1*, *IL1B*, *PTGS1*, *CDKN2A*, *IL1R1*, *CCL2*, *ADA*, *IL6*, *CSF2*, *MMP1*, *and MMP9)* were involved in all the above indicated biological processes. For these functions more over-expressed genes were found when compared to under-expressed ones as shown by the unbalanced ratios (S7 Table). A large number of DEGs were also associated to several physiological system functions and molecular and cellular functions. For cell skeleton (466 DEGs), cell proliferation (880 DEGs), cell apoptosis (760 DEGs) and signal transduction (755 DEGs) a large number of genes are identical to GeneCards database. On the contrary to the above, for these functions the number of over- and under-expressed genes are similar and the corresponding ratios are close to one (S7 Table).

Based on the results described in S7 Table, the list of the top 10 most significantly over- and under-expressed genes with respect to immune function, inflammation, infection and disease, are listed in S8 Table. Some of these genes and their products have a pivotal role for cells in response to disease as shown by their presence in several of the corresponding above processes (S8 Table). In particular, *CX3CL8* was involved in all of 10 biological processes. Very high proportions are also observed for *CX3CL1* (8/10), *TNF* (8/10), *CXCL6* (5/10), *C3* (9/10), *IL1A* (7/10) and *LGALS9* (6/10) over-expressed. Similarly high proportions are observed for some of the under-expressed genes such as *PTHLH* (6/10) and *TIMP3* (5/10).

S9 Table provides the top 100 most significantly over- and under-expressed genes by log_2_ fold-change in response to 2 μg/mL of LPS treatment. Among the 19 most significantly over-expressed genes (> 3 fold), 7 were associated with inflammation *(CXCL6*, *BCL2A1*, *LGALS9*, *C3*, *BIRC3*, *CFB*, and *CD40*) and 9 out of 19 genes were associated with infection *(CXCL6*, *BCL2A1*, *LGALS9*, *C3*, *BIRC3*, *CFB*, *CD40*, *CTSC* and *TCN1)*. On the contrary, among the six most under-expressed genes (>1.5-fold), no DEGs were known to be related to inflammation.

**Fig 5:**
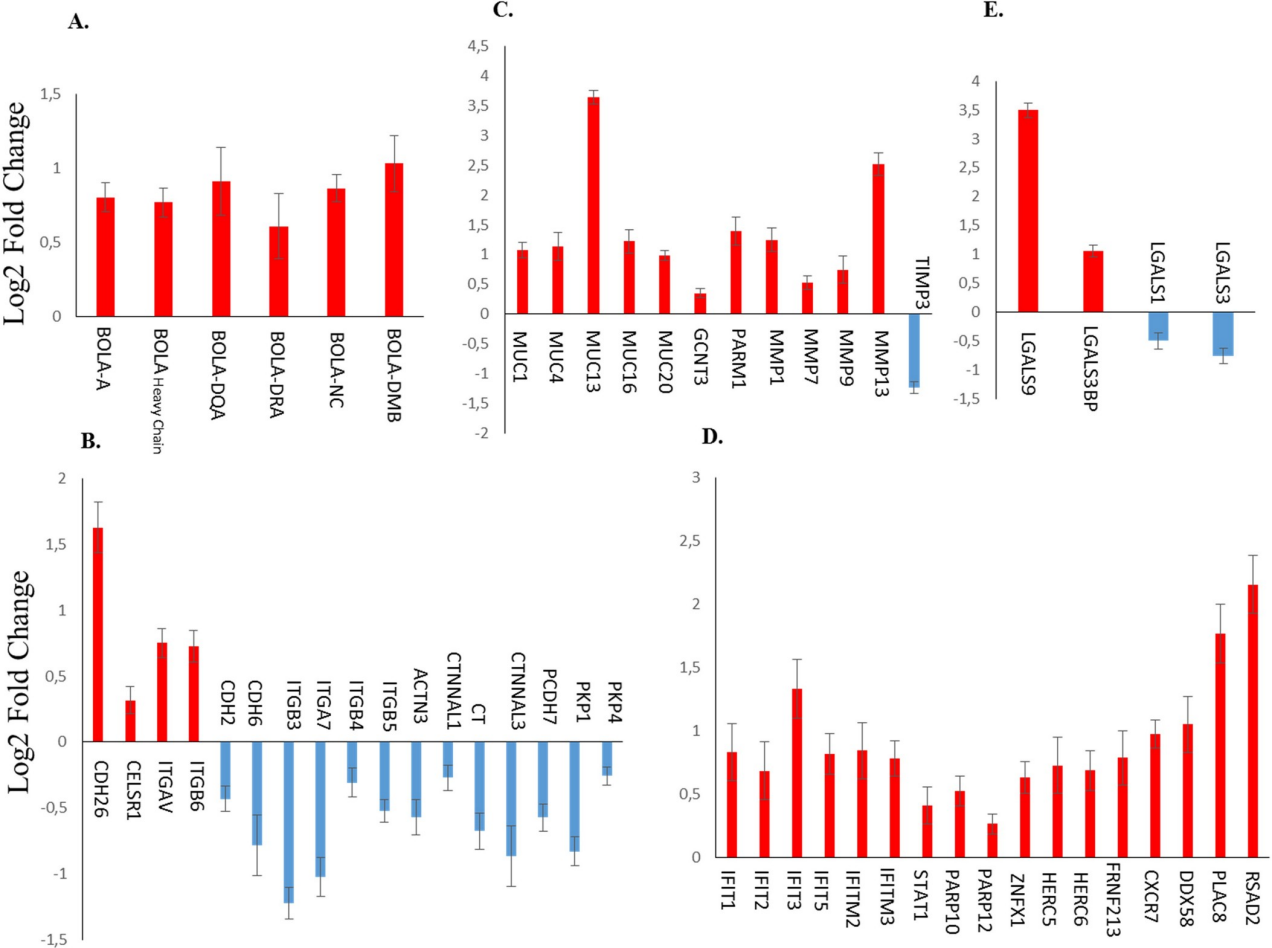
Mean Log2 fold changes (mean ± sem) for DEGs related to implantation and maternal response to the embryo. A. Changes in mRNA expression for genes of the bovine leukocyte antigen molecules (BoLA) family. B. Changes in expression for genes related to cell adhesion. C. Changes in expression for mucins, integrins and metalloproteinase genes. D. Changes in expression for interferon Tau induced genes. E. Changes in expression for genes of the galectin family. All changes significant p < 0.05 from adjusted p value.

Genes of the bovine leukocyte antigen (BoLA) which are part of the Major Histocompatibility Complex (MHC) of cattle were over-expressed. We found that both genes encoding class I (BOLA-A, BOLA-NC), and class II molecules (HLA-DMB, BOLA-DQA1, and BOLA-DRA) were over-expressed after LPS-treatment (Fig 5A, and S1B Fig).

### Genes related to implantation and maternal response to the embryo

A large number of genes encoding for proteins belonging to several families of molecules involved in embryo maternal interactions and implantation were differentially expressed in response to LPS treatment. This includes genes encoding molecules involved in cell structure (actins, actinin), calcium metabolism and membrane properties (calcitonin), cell adhesion (catenins, plakophilin, cadherins, integrins), protection of epithelium (mucins) and enzymes such as Matrix Metallo Peptidase (MMPs) controlling tissue-remodeling.

Four members of the cadherin superfamily which are transmembrane glycoproteins that mediates cell-cell interactions through calcium binding (i.e. *ITGB6*, *CDH26*, *ITGAV* and *CELSR1*) were over-expressed after LPS-treatment. In addition, four integrin-family transcripts (*ITGB3*, *ITGB4*, *ITGB5*, and *ITGA7*) and most of transcripts coding for cell adhesion molecules (for instance, *CTNNA3*, *CTNNAL1*, *CDH2*, *PCDH7*, *CT*, *PKP1*, *PKP4*) showed lower mRNA levels after treatment by LPS (Fig 5B). Furthermore, we found that many genes of the mucin family (*MUC1*, *MUC4*, *MUC13*, *MUC16*, *MUC20*, *and F1MUC1*) and genes of matrix metalloproteinase (MMPs) family (*MMP1*, *MMP7*, *MMP9*, *MMP13*) were strongly over-expressed. Consistent with the above results, their inhibitor *TIMP3* was shown to be strongly under-expressed after LPS-treatment (Fig 5C).

A large number of interferon-τ (IFNT)-induced genes were all over-expressed after LPS-treatment (Fig 5D). These include *IFIT1*, *IFIT2*, *IFIT3*, *IFIT5*, *IFITM2*, *IFITM3*, *PARP12*, *ZNFX1*, *HERC6*, *RNF213*, *CXCR7*, *DDX58*, *PLAC8*, *RSAD2* and *STAT1*. Genes of galectin family were also significantly de-regulated. For instance, Gal-9 (*LGALS-9*), which has been related to response infection is strongly over-expressed. On the contrary, *LGALS-1* and *LGALS-3* were under-expressed in both LPS groups *vs* controls (Fig 5E).

### Genes related to methylation and acetylation

In addition, significant changes in expression for a set genes susceptible to influence the methylation and acetylation profiles of cells were found. Most particularly, MBD4 which binds de-novo methyl transferases (DNMTs) to DNA and a number of genes from the methyl transferase family i.e. METTL9 (methyltransferase like 9), METTL22 (methyltransferase like 22), TRMT1 (TRM1 tRNA methyltransferase 1 homolog) were under-expressed whereas two members of the lysine demethylase family (KDM2 and KDM7) were overexpressed. Concomitantly, three members of the Histone Deacetylase family (i.e. HDAC4, 5 and 6) were under-expressed, whereas the acetyl transferases *(ACAT2)* was over-expressed. Inhibition of HDAC induces transcriptional repression of high copy number genes in breast cancer [39] and ACAT2 gene encode enzymes involved in lipid metabolism [40].

### RT^2^-PCR validation and temporal changes in gene expression induced by LPS

**Fig 6:**
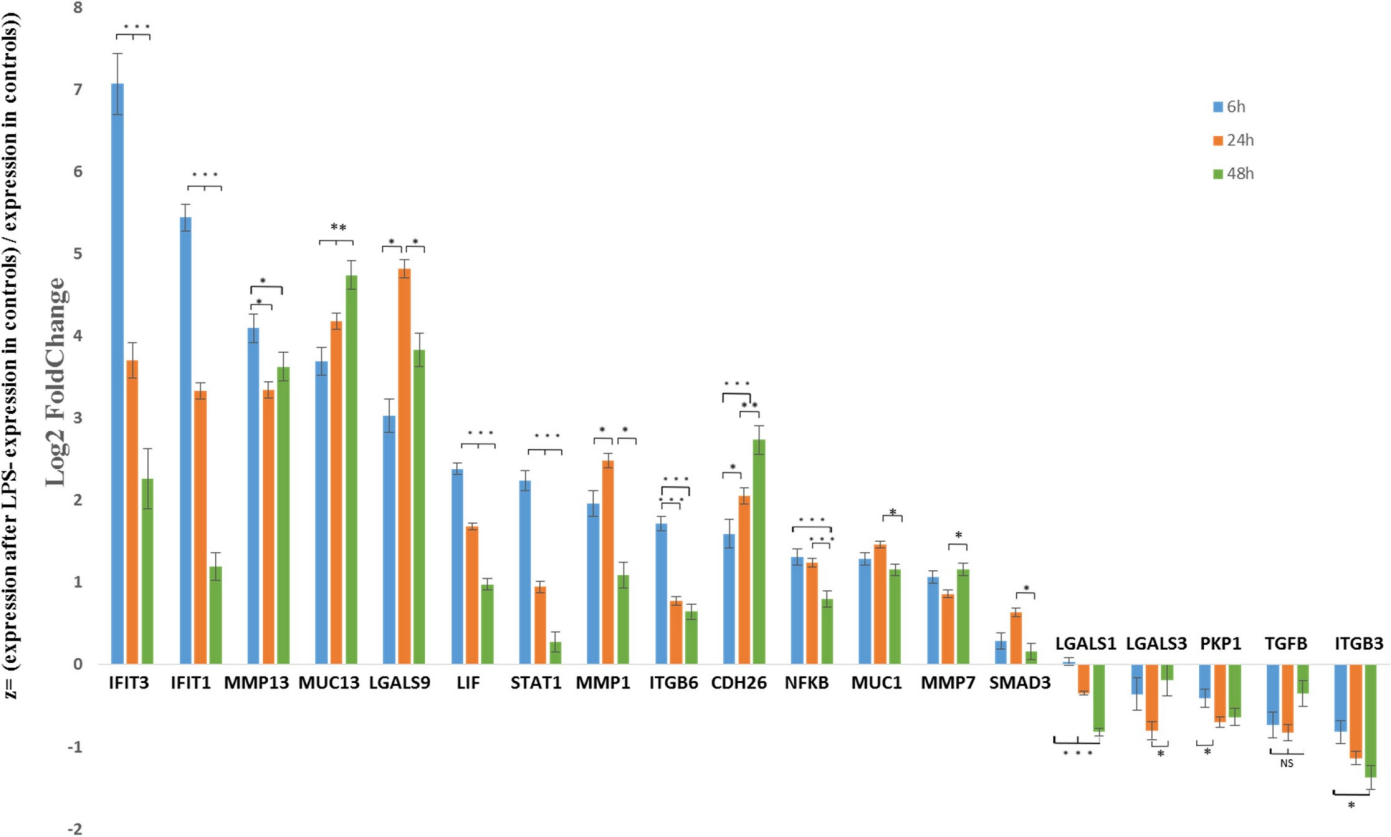
Changes in gene expression from RT^2^-qPCR at 6, 24 and 48 hours following LPS challenge. For each time point, z = (expression after LPS- expression in controls) / expression in controls) for a set of DEGs identified at 24 hours post LPS from RNA sequencing. The order of genes from left to right is organized following the amplitude of fold change at 6 hours from RT^2^-qPCR. NS: not significant, * p < 0.05, ** P < 0.01, *** p < 0.001.

RT^2^-qPCR validation study of 19 differentially expressed genes (over or under expressed) from RNA-seq showed that all tested genes varied significantly and in the same way with the two methods. Notably, all DEGs with an adjusted p value close to 0.05 from RNAseq were confirmed as differentially expressed by the RT^2^-qPCR validation. Moreover, the correlation coefficient between fold changes obtained by RNA-seq and RT^2^-qPCR from samples obtained at 24 hours for the 2 μg/ml LPS dosage was very high with a *r*^*2*^ value close to 1 (Table 1). Gene-expression profiles at 6, 24, and 48 hours revealed differences in patterns between groups of genes. Genes related to acute inflammation and signal transduction pathways such as *IFIT1*, *IFIT3*, *ITGB6*, *NFKB*, *MMP13* and including transcription factors such as *LIF* and *STAT1* expressed a maximum increase at 6 hours and gradually dropped at 24 and 48 hours. For *MUC13*, *CDH26* and *MMP7* gene expression continuously increased from 6 to 48 hours whereas *LGALS9*, *SMD3*, *MUC1 and MMP1* shown a higher expression at 24 hours followed by a decrease. For under-expressed genes, the expression profiles shown that *LGALS1*, *ITGB3* and to a lesser extent *PKP1* were decreasing continuously from 6 to 48 hours, whereas *LGALS3*, and *TGFB* presented low expression at 6 and 24 hours, and were closer to control levels at 48 hours (Fig 6).

**Table 1:** Fold changes obtained by RNA-seq and RT2-qPCR from samples obtained at 24 hours for the 2 μg/ml LPS dosage.

**Gene ID**	**Fold changes (2 μg/ml *vs* control, 24h)**	
	RNA-seq	RT^2^ -qPCR RQ
***STAT1***	1.51	1.93
***LGALS9***	11.25	28.17
***NFKB1***	2.41	2.36
***SMAD3***	1.48	1.56
***MMP1***	12.43	5.20
***MMP7***	1.44	1.82
***MMP13***	5.73	10.17
***LIF***	3.29	3.69
***LGALS1***	-0.71	-0.80
***LGALS3***	-0.59	-0.60
***TGFB2***	0.70	0.73
***ACTB***	1.09	1.04
***TBP***	1.03	1.04
***IFIT1***	1.79	10.07
***IFIT3***	2.53	13.06
***MUC1***	2.08	2.76
***MUC13***	12.55	18.09
***ITGB3***	-0.43	-0.46
***ITGB6***	1.66	1.71
***CDH26***	3.1	4.15
***PKP1***	-0.56	-0.62

R^2^ = 0.97

## Discussion

In a recent study based on the same bEEC model treated with the same concentrations of *E*. *coli* LPS, we previously reported, that the treatment did not alter cell viability and stimulated the proliferation of cells [9]. Despite that the stimulation of proliferation was higher with 8 μg/mL LPS at 72 h after treatment, significant differences in gene expression at 24 hours after treatment between 2 and 8 μg/mL LPS were not observed. In addition, we found numerous changes in protein expression 72 hours after challenge indicating strong alterations of several cell functions [22]. The changes included de-regulation of proteins related to translation, metabolism, glycolysis and related oxidative stress which were over-expressed and down regulation of proteins related to cell structure and adhesion and specific proteins involved in immune-tolerance (such as GAL-1 and TGFβ). Samples from the present experiment were taken at an earlier time than for the proteomic experiment to detect changes in mRNA expression. The pathway analysis of the present study, showing differential gene expression leading to a stimulation of cell metabolism, accompanied by activation of the glycolytic and oxidative stress pathways was fully consistent with the changes observed from our proteomic analysis [22]. The down regulation of cell structure and cell adhesion pathways was also observed from both proteomics [22] and the present RNA sequencing results. When compared to other studies made in the cow [20, 21, 29, 41], human [30] and mouse [42] endometrium, our results fully confirm that a high number of differentially expressed genes relates to immune response, response to infection and inflammation and also to cell metabolism and oxidative stress [22, 43]. Former studies in the cow, reported changes in genes involved in embryo maternal interaction, notably interferon (IFN)-stimulated genes *RSAD2*, *MX2*, *OAS1*, *ISG15*, *BST2;* as well as several molecules involved in the intracellular recognition of pathogens or their ligands, including *IFIH1*, *MDA5*, *DEXH Asp-Glu-X-His box polypeptide 58 (DHX58)*, and up-regulation of a large number of chemokines such as *IL8*, *CCL4*, *CCL20*, *CXCL2*, *CXCL3*, *CSCL10*, *CXCL11*, *CCL3*, *CCL4*, *CCL11*, and *CCL20*. [20]. In addition, gene changes in *MMP1*, *MMP3*, and *MMP13* in response to LPS were also reported [44, 45].

We confirm here changes in expression for most/all these genes. However, results from this data set enlarge the list of gene expression changes corresponding to specific key molecules for embryo-maternal interaction and implantation. As cows previously infected by gram negative bacteria present infertility and high culling rate after infection [1, 4, 46] the significance of the above changes in relation with persistent inflammation and establishment of pregnancy will be discussed.

Our data indicate that epithelial cells are highly sensitive to LPS and activate strongly the corresponding inflammatory pathways. In particular, the toll-like receptor-signaling pathway was significantly affected by LPS treatment, and 19 of the over-expressed DEGs were associated with this pathway including many genes encoding cytokines and chemokines. This is in full agreement with former information showing that LPS provokes the activation of the host’s innate immune response by increasing TLR4- and MyD88-dependent signaling [47]and subsequent expression of pro-inflammatory cytokines and chemokines following binding of TLR4 [22, 47, 48], and activation of JAK/STAT signaling pathway [49] The toll-like receptor signaling pathway activates key molecules to drive immune-related responses towards infectious agents and attracts immune cells by the site of infection [29, 50]. In a first step, this represents one of the main defense mechanisms of the mucosal epithelium. It has also been suggested that the increased expression of cytokine and chemokine genes leads to a persistent presence of immune cells in the endometrium possibly altering implantation [50]. Chemokines can promote or inhibit human trophoblast cell migration and invasion in first-trimester placenta [51]. For instance, Tumor Necrosis Factor alpha (TNF-α) has pleiotropic effects on cell growth, inflammation and innate immunity in the endometrium and is strongly involved in embryo development and implantation [10, 52, 53]. These effects can be either beneficial or detrimental and high concentrations of TNF-α has been reported to be the source of implantation failures and pregnancy loss [54]. Chemokines like CXCL1 and CXCL6 contribute to neutrophil recruitment and are associated with pathways involved in inflammation and apoptosis [55].

From this bEEC *in vitro* model, despite the fact that cells were exposed to relatively low dosages of LPS [9] and for a short time when compared to the in vivo situation, we observed very strong changes in expression of both cytokines and chemokines. The duration of these changes in gene expression should be evaluated further from *in vivo* studies. Such changes may alter the uterine environment and may not be favorable to the transit of spermatozoa and their survival through the female genital tract [56]. However, due to the very delicate immune balance necessary for the establishment of pregnancy [4, 5] and to the very high fold change for some of the genes, it may be speculated that in cows which have been exposed to endometritis, changes in gene expression may also contribute to alter embryo-maternal relationships after infection has disappeared [57]. Undiagnosed persistent inflammation may also represent a limitation in case of embryo transfer.

Complementary to the above mechanisms related to infection and associated immune response, this study shows that many molecules specifically related to embryo development and implantation are strongly de-regulated by LPS. It has been established for a long time that in ruminants, Interferon-τ (IFNT) is the main signal for maternal recognition of pregnancy which prevents prostaglandin F2α induced luteolysis [58]. This molecule is also critical for implantation by regulating the function of a large number of genes called IFNT-induced genes, which are regulated through the STAT-dependent signaling pathway [16, 59], which is activated at the beginning of pregnancy [60]. Activation of this pathway is driven by the developing embryo through secretion of IFNT which induces tyrosine phosphorylation of STAT-1, -2 and -3 [61]. In addition, leukemia inhibitor factor (LIF)-dependent STAT activation is critical for embryo-endometrial interaction and trophoblast invasion [10]. LIF is highly expressed in mouse uterus during receptivity phase and essential for embryo implantation [52]. Without LIF, the downstream signal transduction pathways may be crippled [62, 63]. Decreased expression or complete lack of LIF production is linked to implantation failure [10]. Contrary to what was reported above, the present experiment shows that after a short exposure of endometrial cells to LPS, both *LIF* and *STAT1* genes exhibited a strong increase in mRNA expression at 6 hours post LPS. Despite the amplitude of differential expression for these genes decreased with time, *LIF* mRNA remained strongly overexpressed by 48 hours and many downstream genes such as *MUC1*, *MUC13*, *MMP1*, *MMP7* and *MMP13 w*ere shown to be strongly over-expressed at 48 hours. As reported above for the immune response, these results indicate that it would be useful to study the duration of these changes and their amplitude, and compare them with what is happening in response to a living embryo at the beginning of pregnancy [10]. The LPS-induced changes, if persistent, may also disturb responses of the endometrial cells to IFNT and possibly impair the success of implantation.

In addition to those referred above, our results suggest that LPS activates mechanisms altering specifically cell structure and cell adhesion properties of endometrial epithelial cells which could also be unfavorable to subsequent implantation. Successful embryo implantation requires a subtle regulation of tissue remodeling by adhesion molecules (cadherins, integrins, selectins, and MMPs) [64, 65]. The members of the cadherin superfamily mediate cell-cell interactions though calcium binding, and any possible changes could impair implantation [66]. Our results clearly show that all DEGs encoding proteins involved in cell adhesion and actin cytoskeleton which are important for the binding of growth factors to their respective receptors were down-regulated after LPS challenge. Furthermore, MMPs and its tissue inhibitors of MMPs (TIMPs) are enzymes that mediate immune response to infection, and are involved in remodeling of the extracellular matrix (ECM) during pregnancy and parturition [67]. Their activities must be well balanced for successful implantation. Decreased expression of MMP2 and TIMP3 were detected in the endometrium of women experiencing implantation failure [68, 69]. The significance of such changes should be more documented from further functional studies in the cow. However, since TIMP3 exerts a function as the major modulator of extracellular matrix degradation during implantation [70, 71], the LPS-induced down-regulation of *TIMP3* mRNA, as observed here may be unfavorable to interactions between the conceptus and the endometrium.

We also found that a group of genes coding for proteins belonging to the galectin (Gal) family was de-regulated by LPS suggesting that LPS may alter early embryo–maternal communication [72, 73]. *In vivo* and *in vitro* studies showed that Gal-1, 3 and 9 play a crucial role in the cell proliferation, adhesion processes, and modulation of innate and adaptive immunity, pro-inflammation and/or regulation of immunosuppressive activity. Gal-1 is required for establishing an immune-privileged local environment for implantation and early fetal development which relates especially from its immunosuppressive activities and key role for maternal-fetal tolerance as documented in humans and rodents [74–76]. Gal-1 is up-regulated during normal pregnancy and expressed also in human preimplantation embryos. This protein stimulates FOXP3 which controls the differentiation of T naive cells into T regulatory cells [77]. Gal-1 also positively regulates the expression of human leukocyte antigen G (HLA-G) which inhibits NK cells and modulates cytokine secretion to control trophoblast cell invasion and to maintain a local immunotolerance during implantation [13, 14]. Probably as the result of the above, low expression in the endometrium has been associated with an increased frequency of early pregnancy failures and miscarriages [78, 79]. It has been reported that Gal-1 was present in the bovine endometrium, mainly in the lamina propria [80], but the exact roles of Gal-1 and how it regulates *BOLA* gene expression during pregnancy is not known in this species. The decreased expression of Gal-1 mRNA as observed here corroborates the decreased expression of Gal-1 protein following LPS challenge [22], which has been observed at a later stage.

In the cow, Gal-3 has been identified to be expressed in the uterus, cervix, oviduct, atretic follicles, and regressing corpus luteum [81]. Gal-3 down-regulation may be involved in the changes observed in cell adhesion as these proteins bind to a large set of molecules including integrins, laminin and fibronectin [82]. Gal-9 is expressed in all uterine cell types, including endometrial epithelia and endothelial cells [76]. Over-expression of Gal-9 associated with the increase of expression of many genes coding for pro-inflammatory cytokines suggest that Gal-9 may control positively the production of these molecules in the absence of immune cells [83].

As mentioned above for other signals, the correlation of the length of the changes in galectins would be useful to evaluate the duration of possible alterations in endometrial function.

In this study, the expression of the *PTHLH* gene encoding parathyroid hormone-like hormone, presented the most significantly decreased expression level following exposure to LPS (S9 Table). PTHLH has been localized in the cyclic endometrium in both the glandular and stromal compartments [84] and exerts multiple biological functions in both normal and pathological conditions. A recent study showed that significantly decreased blastocyst formation rate of Pthlh-depleted embryos, and this effect can be overturned by injection of recombinant PTHLH [85]. TGF-β-dependent signaling activates PTHLH expression by increasing transcription from the P3 promoter through a synergistic interaction of the transcription factors Smad3 and Ets1 [86], and p38 MAPK-dependent signaling controls PTHLH expression in metastatic colon cancer cells [87]. Few studies have investigated the characteristics of its expression following LPS exposure [88], however, the strong under expression observed here is very consistent with the up-stream under expression of TGF-β both contributing to impairments of cell adhesion processes.

Finally, the changes observed in genes susceptible to influence the methylation and acetylation profiles of bEECs suggest that LPS may globally induce lower methylation due to under- expression of a set of methyl transferases concomitant with effects on MBD4 which binds DNMTs to DNA. On the contrary, acetylation may be maintained through under-expression of a set of histone deacetylases and over-expression of numerous acetyl transferases. These results are consistent previous results showing that bacterial infections and viral infections induces de-methylation changes in host cells [89–91] and with the overall methylation patterns observed from Reduced Representation Bisulfite Sequencing [92].

## Conclusion

The high number of DEGs and multiple pathways affected by LPS observed in this study confirms that this molecule affects in a complex manner multiple functions of endometrial epithelial cells. Changes in proliferation, general metabolism, glycolysis, oxidative stress and immune response have been extensively described in the literature. The present study gives more insight in the way LPS affects specifically key molecules and pathways involved in embryo-maternal interactions and immuno-tolerance.

These results indicate clearly that most cellular events necessary at time of implantation for the establishment of a successful crosstalk between the endometrium and the developing embryo are perturbed. In addition, changes in expression for genes that control methylation may be the cause of long term consequences of previous infection on uterine function. These findings provide new insights regarding the mechanisms by which LPS and induced inflammation may alter uterine receptivity. This information showing a multiplicity of differential gene expression patterns in response to LPS treatment suggest to perform additional studies to evaluate further, the persistence of those changes and their functional impact on fertility.

## Supporting information

S1 Fig Overrepresented KEGG pathways in relation with immune response. A. Toll like receptor signaling pathway and B. Antigen processing and presentation pathway. Each rectangle represents a gene in the pathway. The left part of the rectangle corresponds to the comparison between control 24h and control time 0, the middle part corresponds to the comparison between LPS (2 μg/mL) and control 24h, and the right part between LPS (8 μg/mL) and control 24h. Colors indicate that the gene was either (adj P < 0.05) under-expressed (green) or over-expressed (red) whereas grey color indicates no change (adj P > 0.05).
(TIF)

S1 Table Summary of RNA quality and RNA-seq reads mapping to reference genome. (DOCX)

S2 Table Distribution of the GO functional terms predicted. (DOCX)

S3 Table List of overrepresented Go terms. (DOCX)

S4 Table List of underrepresented Go terms. (DOCX)

S5 Table List of overrepresented Kegg pathways. (DOCX)

S6 Table List of underrepresented Kegg pathways. (DOCX)

S7 Table Differentially expressed genes related to diseases and responses. (DOCX)

S8 Table Top 10 most significantly over- and under-expressed DEGs associated to each responses and diseases. (DOCX)

S9 Table List of the top 100 over-expressed and under-expressed transcripts in bEEC after challenged with LPS. (DOCX)
