# Supplementary figures and images for: Differential gene expression in bovine endometrial epithelial cells after challenge with LPS; specific implications for genes involved in embryo maternal interactions
Source: PLoS One. 2019 Sep 5;14(9):e0222081. doi: 10.1371/journal.pone.0222081 (PMC6728075; doi:10.1371/journal.pone.0222081)

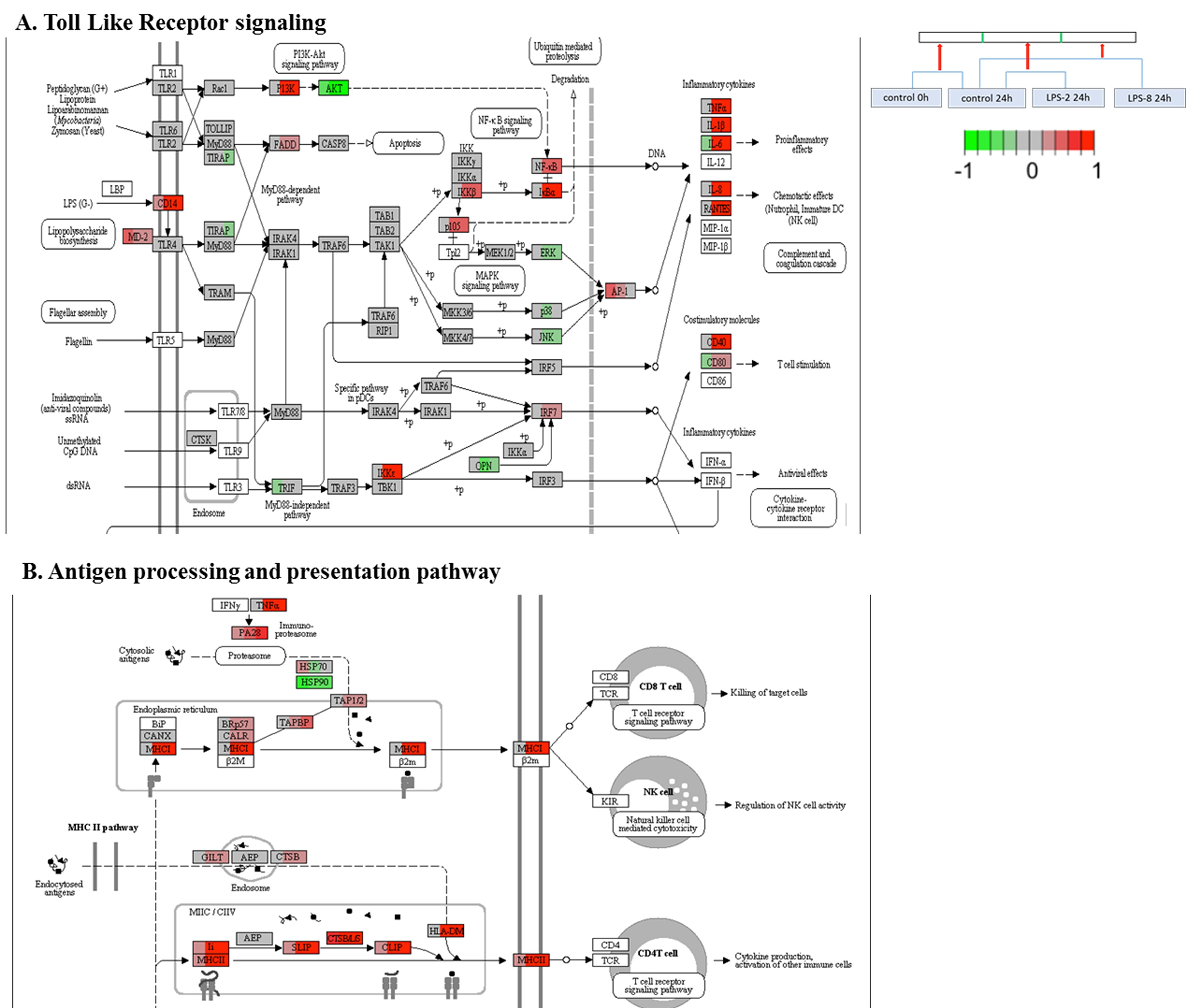

Supplement: S1 Fig — A. Toll like receptor signaling pathway and B. Antigen processing and presentation pathway. Each rectangle represents a gene in the pathway. The left part of the rectangle corresponds to the comparison between control 24h and control time 0, the middle part corresponds to the comparison between LPS (2 μg/mL) and control 24h, and the right part between LPS (8 μg/mL) and control 24h. Colors indicate that the gene was either (adj P < 0.05) under-expressed (green) or over-expressed (red) whereas grey color indicates no change (adj P > 0.05). (TIF) [file pone.0222081.s001.tif]
