# Supplementary material for: Differential gene expression in bovine endometrial epithelial cells after challenge with LPS; specific implications for genes involved in embryo maternal interactions
Source: PLoS One. 2019 Sep 5;14(9):e0222081. doi: 10.1371/journal.pone.0222081 (PMC6728075; doi:10.1371/journal.pone.0222081)
Supplement: S1 Table — (DOCX) [file pone.0222081.s002.docx]

**Supplementary S1 Table: Summary of RNA quality and RNA-seq reads mapping to reference genome**

| Samples  Cow | RNA (RIN) | Concentration | LPS (µg/ml) | Total reads | TrimmedReads.  Both Surviving | MappedReads.  Unique | % Unique mapped reads |
| --- | --- | --- | --- | --- | --- | --- | --- |
|  |  | (ng/µl) |  | (× 10^6^) | (× 10^6^) | (× 10^6^) |  |
| No.1 | 10 | 1361 | T0 * | 28.49 | 28.43 | 26.89 | 94.58 |
|  | 10 | 1037 | C0 ** | 27.58 | 27.52 | 25.95 | 94.29 |
|  | 9.9 | 805 | 2 | 28.16 | 28.10 | 26.53 | 94.41 |
|  | 9.9 | 946 | 8 | 29.82 | 29.75 | 28.03 | 94.22 |
| No.2 | 9.1 | 1018 | T0 * | 29.99 | 29.92 | 27.56 | 92.11 |
|  | 8.8 | 1081 | C0 ** | 30.08 | 29.98 | 26.82 | 89.46 |
|  | 9.0 | 1450 | 2 | 29.19 | 29.12 | 26.39 | 90.63 |
|  | 8.9 | 1417 | 8 | 28.78 | 28.71 | 26.75 | 93.17 |
| No.3 | 10 | 1580 | T0 * | 26.11 | 26.05 | 24.58 | 94.36 |
|  | 9.9 | 1227 | C0 ** | 25.30 | 25.23 | 23.81 | 94.37 |
|  | 9.8 | 1405 | 8 | 24.97 | 24.89 | 23.28 | 93.53 |
|  | 9.8 | 1096 | 8 | 31.54 | 31.46 | 29.63 | 94.18 |
| Average |  |  |  | 28.33 ± 2.03 | 28.26 ± 2.03 | 26.35 ± 1.96 |  |
| Overall |  |  |  | 340.01 | 339.16 | 316.21 |  |

(T0 * = Time 0 control. no LPS; C0 ** = 24h control no LPS
