# Supplementary material for: Differential gene expression in bovine endometrial epithelial cells after challenge with LPS; specific implications for genes involved in embryo maternal interactions
Source: PLoS One. 2019 Sep 5;14(9):e0222081. doi: 10.1371/journal.pone.0222081 (PMC6728075; doi:10.1371/journal.pone.0222081)
Supplement: S2 Table — (DOCX) [file pone.0222081.s003.docx]

**Supplementary S2 Table: Distribution of the GO functional terms predicted**

| Go terms | Go term branch | | | |
| --- | --- | --- | --- | --- |
|  |  | **BP** | **CC** | **MF** |
| **Overrepresented** | Total 274 | 214 (78.1%) | 15 (5.5%) | 45 (16.4%) |
|  | Top 100 | 76 (76%) | 9 (9%) | 15 (15%) |
|  | Top 50 | 32 (64%) | 8 (16%) | 10 (20%) |
|  |  |  |  |  |
| **Underrepresented** | Total 210 | 134 (64%) | 52 (25%) | 24 (11%) |
|  | Top 100 | 59 (59%) | 35 (35%) | 6 (6%) |
|  | Top 50 | 31 (62%) | 15 (30%) | 4 (8%) |

GO = Gene Ontology; BP = Biological Process; CC = Cellular Component; MF = Molecular Function
