# Supplementary material for: Differential gene expression in bovine endometrial epithelial cells after challenge with LPS; specific implications for genes involved in embryo maternal interactions
Source: PLoS One. 2019 Sep 5;14(9):e0222081. doi: 10.1371/journal.pone.0222081 (PMC6728075; doi:10.1371/journal.pone.0222081)
Supplement: S3 Table — (DOCX) [file pone.0222081.s004.docx]

**Supplementary S3 Table: List of overrepresented Go terms**

| GO ID | Term | Rapport.Count | Adj. *P* value |
| --- | --- | --- | --- |
| **GO:0002376** | immune system process : BP | 82 | 4.54471E-24 |
| **GO:0006955** | immune response : BP | 60 | 3.85692E-21 |
| **GO:0050896** | response to stimulus : BP | 128 | 4.2352E-15 |
| **GO:0006954** | inflammatory response : BP | 28 | 6.24441E-12 |
| **GO:0006952** | defense response : BP | 40 | 9.71582E-12 |
| **GO:0048002** | antigen processing and presentation of peptide antigen : BP | 13 | 2.36839E-11 |
| **GO:0009611** | response to wounding : BP | 35 | 3.08474E-11 |
| **GO:0019882** | antigen processing and presentation : BP | 20 | 6.83453E-11 |
| **GO:0002474** | antigen processing and presentation of peptide antigen via MHC class I : BP | 10 | 1.66341E-09 |
| **GO:0005576** | extracellular region : CC | 87 | 7.38849E-09 |
| **GO:0009605** | response to external stimulus : BP | 44 | 1.26125E-08 |
| **GO:0044421** | extracellular region part : CC | 53 | 1.43102E-08 |
| **GO:0006950** | response to stress : BP | 70 | 1.58786E-08 |
| **GO:0002682** | regulation of immune system process : BP | 32 | 3.83532E-08 |
| **GO:0009986** | cell surface : CC | 25 | 6.60735E-08 |
| **GO:0002684** | positive regulation of immune system process : BP | 26 | 8.83182E-08 |
| **GO:0051704** | multi-organism process : BP | 28 | 2.15135E-07 |
| **GO:0005615** | extracellular space : CC | 37 | 2.24526E-07 |
| **GO:0048518** | positive regulation of biological process : BP | 78 | 3.47144E-07 |
| **GO:0009607** | response to biotic stimulus : BP | 26 | 5.43519E-07 |
| **GO:0005886** | plasma membrane : CC | 100 | 8.13919E-07 |
| **GO:0050867** | positive regulation of cell activation : BP | 17 | 1.32089E-06 |
| **GO:0009897** | external side of plasma membrane : CC | 17 | 1.42246E-06 |
| **GO:0042221** | response to chemical stimulus : BP | 47 | 1.46422E-06 |
| **GO:0050865** | regulation of cell activation : BP | 19 | 1.91269E-06 |
| **GO:0002694** | regulation of leukocyte activation : BP | 18 | 4.20362E-06 |
| **GO:0042379** | chemokine receptor binding : MF | 11 | 4.30922E-06 |
| **GO:0008009** | chemokine activity : MF | 11 | 4.30922E-06 |
| **GO:0002696** | positive regulation of leukocyte activation : BP | 16 | 4.48624E-06 |
| **GO:0048522** | positive regulation of cellular process : BP | 67 | 5.3635E-06 |
