# Supplementary material for: Differential gene expression in bovine endometrial epithelial cells after challenge with LPS; specific implications for genes involved in embryo maternal interactions
Source: PLoS One. 2019 Sep 5;14(9):e0222081. doi: 10.1371/journal.pone.0222081 (PMC6728075; doi:10.1371/journal.pone.0222081)
Supplement: S4 Table — (DOCX) [file pone.0222081.s005.docx]

**Supplementary S4 Table: List of underrepresented Go terms**

| GO-ID | Term | Rapport.Count | Adj *P* value |
| --- | --- | --- | --- |
| **GO:0005856** | cytoskeleton : CC | 55 | 5.28718E-09 |
| **GO:0044430** | cytoskeletal part : CC | 37 | 3.02603E-06 |
| **GO:0005488** | binding : MF | 366 | 4.48796E-05 |
| **GO:0007167** | enzyme linked receptor protein signaling pathway : BP | 19 | 7.59046E-05 |
| **GO:0043228** | non-membrane-bounded organelle : CC | 72 | 0.000248378 |
| **GO:0043232** | intracellular non-membrane-bounded organelle : CC | 72 | 0.000248378 |
| **GO:0006996** | organelle organization : BP | 46 | 0.000291421 |
| **GO:0007010** | cytoskeleton organization : BP | 19 | 0.000399941 |
| **GO:0048856** | anatomical structure development : BP | 64 | 0.000411477 |
| **GO:0003779** | actin binding : MF | 16 | 0.00063688 |
| **GO:0005515** | protein binding : MF | 212 | 0.000792798 |
| **GO:0005737** | cytoplasm : CC | 183 | 0.000827558 |
| **GO:0015630** | microtubule cytoskeleton : CC | 21 | 0.000903171 |
| **GO:0048514** | blood vessel morphogenesis : BP | 13 | 0.001648771 |
| **GO:0005622** | intracellular : CC | 273 | 0.00168708 |
| **GO:0051493** | regulation of cytoskeleton organization : BP | 9 | 0.001786002 |
| **GO:0048519** | negative regulation of biological process : BP | 48 | 0.001929167 |
| **GO:0022402** | cell cycle process : BP | 19 | 0.002039222 |
| **GO:0007275** | multicellular organismal development : BP | 68 | 0.002103616 |
| **GO:0032501** | multicellular organismal process : BP | 88 | 0.002148675 |
| **GO:0050793** | regulation of developmental process : BP | 24 | 0.002219403 |
| **GO:0032502** | developmental process : BP | 74 | 0.002235268 |
| **GO:0065007** | biological regulation : BP | 176 | 0.002430354 |
| **GO:0016043** | cellular component organization : BP | 65 | 0.002456667 |
| **GO:0051494** | negative regulation of cytoskeleton organization : BP | 6 | 0.002536356 |
| **GO:0007169** | transmembrane receptor protein tyrosine kinase signaling pathway : BP | 12 | 0.002941096 |
