# Supplementary material for: Differential gene expression in bovine endometrial epithelial cells after challenge with LPS; specific implications for genes involved in embryo maternal interactions
Source: PLoS One. 2019 Sep 5;14(9):e0222081. doi: 10.1371/journal.pone.0222081 (PMC6728075; doi:10.1371/journal.pone.0222081)
Supplement: S5 Table — (DOCX) [file pone.0222081.s006.docx]

**Supplementary S5 Table: List of overrepresented Kegg pathways**

| KEGG category | Name | Rapport.Count  genes | Adj *P* value | Description |
| --- | --- | --- | --- | --- |
| **04622** | RIG-I-like receptor signaling pathway | 18 | 4.61754E-08 | Organismal Systems;  Immune system |
| **04621** | NOD-like receptor signaling pathway | 16 | 4.3057E-07 | Organismal Systems; |
| **04623** | Cytosolic DNA-sensing pathway | 13 | 1.02229E-05 | Immune system |
| **04210** | Apoptosis | 18 | 1.02726E-05 | Detecting various pathogens and generating innate immune |
| **04620** | Toll-like receptor signaling pathway | 19 | 1.08754E-05 | Pathogen recognition by TLRs provokes rapid activation of innate immunity by inducing production of proinflammatory cytokines and upregulation of costimulatory molecules |
| **05332** | Graft-versus-host disease | 10 | 2.64083E-05 | Human Diseases; Immune |
| **04060** | Cytokine-cytokine receptor interaction | 25 | 0.000431049 | diseases |
| **04612** | Antigen processing and presentation | 12 | 0.000611082 | Signaling molecules and |
| **04062** | Chemokine signaling pathway | 23 | 0.000962861 | interaction |
| **04940** | Type I diabetes mellitus | 9 | 0.001882398 | Organismal Systems; |
| **05330** | Allograft rejection | 8 | 0.003091343 | Immune system |
