# Supplementary material for: Differential gene expression in bovine endometrial epithelial cells after challenge with LPS; specific implications for genes involved in embryo maternal interactions
Source: PLoS One. 2019 Sep 5;14(9):e0222081. doi: 10.1371/journal.pone.0222081 (PMC6728075; doi:10.1371/journal.pone.0222081)
Supplement: S6 Table — (DOCX) [file pone.0222081.s007.docx]

**Supplementary S6 Table: List of underrepresented Kegg pathways**

| Kegg category | Name | Rapport.Count  genes | Adj. P value | Description |
| --- | --- | --- | --- | --- |
| **04810** | Regulation of actin cytoskeleton | 20 | 0.0002517 | Cellular Processes; Cell motility |
| **05412** | Arrhythmogenic right ventricular cardiomyopathy (ARVC) | 11 | 0.0003971 | Human Diseases; Cardiovascular diseases |
| **04350** | TGF-beta signaling pathway | 12 | 0.0004359 | Signal transduction. structurally related secreted cytokines |
| **05200** | Pathways in cancer | 26 | 0.0005054 |  |
| **04510** | Focal adhesion | 18 | 0.0008966 | Cellular Processes; Cellular commiunity |
| **04010** | MAPK signaling pathway | 21 | 0.0045430 | Signal transduction |
| **05210** | Colorectal cancer | 9 | 0.0131922 | Cancers |
| **00760** | Nicotinate and nicotinamide metabolism | 5 | 0.0166668 | Metabolism; Metabolism of cofactors and vitamins |
| **00520** | Amino sugar and nucleotide sugar metabolism | 8 | 0.0193217 | Metabolism; Carbohydrate metabolism |
| **04910** | Insulin signaling pathway | 11 | 0.0245931 | Leading to activation of glycogen synthase (GYS) |
| **05410** | Hypertrophic cardiomyopathy (HCM) | 8 | 0.0356878 | Cardiovascular diseases |
