# Supplementary material for: Differential gene expression in bovine endometrial epithelial cells after challenge with LPS; specific implications for genes involved in embryo maternal interactions
Source: PLoS One. 2019 Sep 5;14(9):e0222081. doi: 10.1371/journal.pone.0222081 (PMC6728075; doi:10.1371/journal.pone.0222081)
Supplement: S7 Table — (DOCX) [file pone.0222081.s008.docx]

**Supplementary S7 Table: Differentially expressed genes related to diseases and responses**

|  | **Common DEGs** | **Over -expressed** | **Under -expressed** | **Ratio (Over/under)** |
| --- | --- | --- | --- | --- |
| **Acute inflammation** | 410 | 262 | 148 | 1.77 |
| **Innate immune** | 441 | 291 | 150 | 1.94 |
| **Immune tolerance in pregnancy** | 120 | 83 | 37 | 2.24 |
| **Allergy** | 153 | 108 | 45 | 2.40 |
| **Embryo Implantation** | 118 | 61 | 57 | 1.07 |
| **Cell adhesion** | 626 | 316 | 310 | 1.02 |
| **Cell apoptosis** | 760 | 420 | 340 | 1.24 |
| **Cell skeleton** | 466 | 225 | 241 | 0.93 |
| **Cell proliferation** | 880 | 464 | 416 | 1.12 |
| **Signal transduction** | 755 | 391 | 364 | 1.07 |
