# Supplementary material for: Differential gene expression in bovine endometrial epithelial cells after challenge with LPS; specific implications for genes involved in embryo maternal interactions
Source: PLoS One. 2019 Sep 5;14(9):e0222081. doi: 10.1371/journal.pone.0222081 (PMC6728075; doi:10.1371/journal.pone.0222081)
Supplement: S8 Table — (DOCX) [file pone.0222081.s009.docx]

**Supplementary S8 Table: Top 10 most significantly over- and under-expressed DEGs associated to each responses and diseases**

| **Acute inflammation** | | **Innate immune** | | **Embryo implantation** | | **Immune tolerance in pregnancy** | | **Allergy** | |
| --- | --- | --- | --- | --- | --- | --- | --- | --- | --- |
| Genes | Log2 FC | Genes | Log2 FC | Genes | Log2 FC | Genes | Log2 FC | Genes | Log2 FC |
| **Over-expressed** | | | | | | | | | |
| *C3* | 4.55 | *CXCL6* | 6.36 | *C3* | 4.55 | *C3* | 4.55 | *C3* | 4.55 |
| *TCN1* | 3.90 | *C3* | 4.55 | *CXCL8* | 3.36 | *SLC5A5* | 3.80 | *MUC13* | 3.65 |
| *LGALS9* | 3.50 | *TCN1* | 3.90 | *CX3CL1* | 3.33 | *LGALS9* | 3.50 | *LGALS9* | 3.50 |
| *CXCL8* | 3.36 | *SLC5A5* | 3.80 | *TNF* | 2.86 | *CXCL8* | 3.36 | *CXCL8* | 3.36 |
| *CX3CL1* | 3.33 | *MUC13* | 3.65 | *IL1A* | 2.74 | *CCL5* | 3.16 | *CTSC* | 3.33 |
| *CCL5* | 3.16 | *CXCL8* | 3.36 | *IL1B* | 2.40 | *TNF* | 2.86 | *CX3CL1* | 3.33 |
| *CFB* | 3.07 | *CTSC* | 3.33 | *GPX3* | 2.34 | *IL1A* | 2.74 | *CCL5* | 3.16 |
| *TNF* | 2.86 | *CX3CL1* | 3.33 | *PLAT* | 2.22 | *CD40* | 2.61 | *TNF* | 2.86 |
| *IL1A* | 2.74 | *CXCL3* | 3.24 | *CXCR4* | 2.12 | *IL1B* | 2.40 | *IL1A* | 2.74 |
| *ARRB1* | 2.64 | *CCL5* | 3.16 | *CST3* | 2.11 | *PLAT* | 2.22 | *TMPRSS2* | 2.63 |
| **Under-expressed** | | | | | | | | | |
| *KITLG* | -0.98 | *PLXDC2* | -0.94 | *SLC2A3* | -0.75 | *WDR60* | -0.71 | *CCDC114* | -0.65 |
| *F5* | -1.00 | *KITLG* | -0.98 | *LGALS3* | -0.76 | *CAV1* | -0.71 | *KRT18* | -0.75 |
| *TMEM37* | -1.03 | *PEG10* | -0.98 | *IL1R1* | -0.84 | *AKR1B1* | -0.72 | *LGALS3* | -0.76 |
| *IL12RB2* | -1.08 | *PCTP* | -0.98 | *CTGF* | -0.84 | *PPARGC1A* | -0.75 | *IL1R1* | -0.84 |
| *ENPP1* | -1.11 | *ATP6V1B1* | -0.99 | *FLT1* | -0.94 | *SLC2A3* | -0.84 | *CTNNA3* | -0.87 |
| *BPI* | -1.11 | *F5* | -1.00 | *KITLG* | -0.98 | *IL1R1* | -0.94 | *KITLG* | -0.98 |
| *TIMP3* | -1.24 | *IL12RB2* | -1.08 | *F5* | -1.00 | *FLT1* | -0.95 | *F5* | -1.00 |
| *LOX* | -1.25 | *FGFR2* | -1.10 | *FGFR2* | -1.10 | *SLC40A1* | -1.00 | *TMEM37* | -1.03 |
| *TNXB* | -1.33 | *BPI* | -1.11 | *FST* | -1.17 | *F5* | -1.11 | *IL12RB2* | -1.08 |
| *PTHLH* | -1.92 | *RGS6* | -1.29 | *TIMP3* | -1.24 | *ENPP1* | -1.11 | *BPI* | -1.11 |
|  |  |  |  |  |  |  |  |  |  |
| **Cell adhesion** | | **Cell apoptosis** | | **Cell skeleton** | | **Cell proliferation** | | **Signal transduction** | |
| Genes | Log2 FC | Genes | Log2 FC | Genes | Log2 FC | Genes | Log2 FC | Genes | Log2 FC |
| **Over-expressed** | | | | | | | | | |
| *CXCL6* | 6.36 | *CXCL6* | 6.359706 | *C3* | 4.55 | *CXCL6* | 6.36 | *CXCL6* | 6.36 |
| *C3* | 4.55 | *BCL2A1* | 4.217028 | *SLC5A5* | 3.80 | *C3* | 4.55 | *C3* | 4.55 |
| *LGALS9* | 3.50 | *TCN1* | 3.899501 | *CXCL8* | 3.36 | *BCL2A1* | 4.22 | *CXCL8* | 3.36 |
| *CXCL8* | 3.36 | *SLC5A5* | 3.795434 | *CTSC* | 3.33 | *SLC5A5* | 3.80 | *CX3CL1* | 3.33 |
| *CX3CL1* | 3.33 | *LGALS9* | 3.498249 | *CFB* | 3.07 | *LGALS9* | 3.50 | *CXCL3* | 3.24 |
| *CCL5* | 3.16 | *CXCL8* | 3.359399 | *TNF* | 2.86 | *CXCL8* | 3.36 | *CCL5* | 3.16 |
| *TNF* | 2.86 | *CTSC* | 3.326993 | *IL1A* | 2.74 | *CTSC* | 3.33 | *TNF* | 2.86 |
| *IL1A* | 2.74 | *CX3CL1* | 3.325303 | *CD40* | 2.61 | *CX3CL1* | 3.33 | *IL1A* | 2.74 |
| *CD40* | 2.61 | *CCL5* | 3.160256 | *MMP13* | 2.52 | *CXCL3* | 3.24 | *PKD2L1* | 2.66 |
| *BIRC3* | 2.53 | *TNF* | 2.863907 | *IL1B* | 2.40 | *CCL5* | 3.16 | *ARRB1* | 2.64 |
| **Under-expressed** | | | | | | | | | |
| *ADAMTS12* | -1.11 | *TSPAN7* | -1.11 | *DKK2* | -1.07 | *ENPP1* | 1.11 | *FGFR2* | -1.11 |
| *FST* | -1.17 | *ENPP1* | -1.11 | *FGFR2* | -1.10 | *FST* | 1.17 | *TSPAN7* | -1.17 |
| *TIMP3* | -1.24 | *BPI* | -1.11 | *TSPAN7* | -1.11 | *TIMP3* | 1.24 | *FST* | -1.24 |
| *LOX* | -1.25 | *TIMP3* | -1.24 | *ENPP1* | -1.11 | *LOX* | 1.25 | *LOX* | -1.25 |
| *CLDN8* | -1.33 | *LOX* | -1.25 | *FST* | -1.17 | *KMO* | 1.31 | *RASL11B* | -1.31 |
| *TNXB* | -1.33 | *RGS6* | -1.29 | *LOX* | -1.25 | *CLDN8* | 1.33 | *RGS6* | -1.33 |
| *KRT5* | -1.40 | *KMO* | -1.31 | *TNXB* | -1.33 | *TNXB* | 1.33 | *CLDN8* | -1.33 |
| *CYP26A1* | -1.76 | *KRT5* | -1.40 | *KRT5* | -1.40 | *KRT5* | 1.40 | *FGF13* | -1.40 |
| *ECM2* | -1.81 | *FGF13* | -1.71 | *CYP26A1* | -1.76 | *CYP26A1* | 1.76 | *CYP26A1* | -1.76 |
| *PTHLH* | -1.92 | *PTHLH* | -1.92 | *PTHLH* | -1.92 | *PTHLH* | 1.92 | *PTHLH* | -1.92 |
