# Supplementary material for: Differential gene expression in bovine endometrial epithelial cells after challenge with LPS; specific implications for genes involved in embryo maternal interactions
Source: PLoS One. 2019 Sep 5;14(9):e0222081. doi: 10.1371/journal.pone.0222081 (PMC6728075; doi:10.1371/journal.pone.0222081)
Supplement: S9 Table — (DOCX) [file pone.0222081.s010.docx]

**Supplementary S9 Table: List of the top 100 over-expressed and under-expressed transcripts in bEEC after challenged with LPS**

| **Over-expressed genes** | | | | **Under-expressed genes** | | | |
| --- | --- | --- | --- | --- | --- | --- | --- |
| **Ensembl_gene_id** | **Gene_name** | **log2FC** | **padj** | **Ensembl_gene_id** | **Gene_name** | **log2FC** | **padj** |
| ENSBTAG00000009812 | *CXCL6* | 6.36 | 0.00E+00 | ENSBTAG00000000460 | *SYTL2* | -0.76 | 8.71E-07 |
| ENSBTAG00000007449 | *HS3ST1* | 4.69 | 2.56E-154 | ENSBTAG00000004599 | *TAGLN3* | -0.76 | 4.13E-03 |
| ENSBTAG00000017280 | *C3* | 4.55 | 8.56E-125 | ENSBTAG00000035144 | *PLCXD1* | -0.76 | 8.83E-05 |
| ENSBTAG00000000735 | *BCL2A1* | 4.22 | 2.15E-204 | ENSBTAG00000034384 | *VWA3B* | -0.77 | 6.36E-03 |
| ENSBTAG00000010433 | *M-SAA3.2* | 4.02 | 1.20E-67 | ENSBTAG00000047827 | *RENBP* | -0.77 | 7.81E-07 |
| ENSBTAG00000020580 | *TCN1* | 3.90 | 7.09E-157 | ENSBTAG00000007379 | *ALK* | -0.78 | 7.21E-03 |
| ENSBTAG00000015830 | *SLC5A5* | 3.80 | 7.78E-120 | ENSBTAG00000009849 | *PLAC8* | -0.78 | 5.20E-14 |
| ENSBTAG00000037558 | *GRO1* | 3.79 | 1.93E-67 | ENSBTAG00000032187 | *ZNF697* | -0.78 | 7.81E-06 |
| ENSBTAG00000010382 | *MUC13* | 3.65 | 1.37E-218 | ENSBTAG00000017655 | *PALMD* | -0.78 | 1.11E-07 |
| ENSBTAG00000006846 | *LGALS9* | 3.50 | 8.87E-174 | ENSBTAG00000001899 | *KCNH7* | -0.78 | 2.26E-05 |
| ENSBTAG00000022396 | *SAA3* | 3.43 | 1.67E-47 | ENSBTAG00000012992 | *CDH6* | -0.79 | 5.91E-03 |
| ENSBTAG00000019716 | *CXCL8* | 3.36 | 8.28E-47 | ENSBTAG00000021745 | *RAPSN* | -0.79 | 5.03E-04 |
| ENSBTAG00000011100 | *CTSC* | 3.33 | 3.56E-294 | ENSBTAG00000018164 | *FNDC4* | -0.79 | 1.53E-04 |
| ENSBTAG00000024869 | *CX3CL1* | 3.33 | 7.02E-49 | ENSBTAG00000031278 | *SNPH* | -0.79 | 1.58E-03 |
| ENSBTAG00000037778 | *CXCL3* | 3.24 | 1.24E-43 | ENSBTAG00000008507 | *SPHK1* | -0.80 | 2.03E-09 |
| ENSBTAG00000007191 | *CCL5* | 3.16 | 3.80E-46 | ENSBTAG00000003043 | *GNG2* | -0.80 | 2.85E-19 |
| ENSBTAG00000046158 | *CFB* | 3.07 | 1.43E-107 | ENSBTAG00000004287 | *EVC* | -0.80 | 2.77E-11 |
| ENSBTAG00000048171 | *TAP* | 3.04 | 5.08E-37 | ENSBTAG00000018160 | *LAMA1* | -0.80 | 4.60E-04 |
| ENSBTAG00000008471 | *MX2* | 3.02 | 3.27E-66 | ENSBTAG00000004570 | *PRRX1* | -0.82 | 1.11E-08 |
| ENSBTAG00000017674 | *SCNN1D* | 2.89 | 1.94E-48 | ENSBTAG00000037907 | *DSEL* | -0.82 | 3.20E-04 |
| ENSBTAG00000025471 | *TNF* | 2.86 | 1.16E-35 | ENSBTAG00000019870 | *SLC14A1* | -0.83 | 1.40E-03 |
| ENSBTAG00000016683 | *BIKBA* | 2.79 | 2.84E-71 | ENSBTAG00000004490 | *TRIM31* | -0.83 | 5.95E-08 |
| ENSBTAG00000010349 | *IL1A* | 2.74 | 2.26E-30 | ENSBTAG00000034206 | *EMCN* | -0.83 | 3.52E-05 |
| ENSBTAG00000010742 | *PKD2L1* | 2.66 | 2.22E-58 | ENSBTAG00000000835 | *BCAS1* | -0.83 | 3.46E-03 |
| ENSBTAG00000020485 | *ARRB1* | 2.64 | 2.92E-29 | ENSBTAG00000000841 | *LZTS1* | -0.83 | 1.11E-04 |
| ENSBTAG00000009132 | *TMPRSS2* | 2.63 | 4.69E-56 | ENSBTAG00000005273 | *IL1R1* | -0.84 | 8.97E-06 |
| ENSBTAG00000020736 | *CD40* | 2.61 | 5.50E-89 | ENSBTAG00000004222 | *SAT2* | -0.84 | 2.76E-03 |
| ENSBTAG00000024918 | *BIRC3* | 2.53 | 2.47E-110 | ENSBTAG00000006367 | *CTGF* | -0.84 | 4.53E-10 |
| ENSBTAG00000015059 | *MMP13* | 2.52 | 7.88E-36 | ENSBTAG00000011647 | *SLC25A15* | -0.85 | 1.14E-09 |
| ENSBTAG00000001321 | *IL1B* | 2.40 | 1.56E-23 | ENSBTAG00000018703 | *OSTN* | -0.85 | 4.57E-04 |
| ENSBTAG00000011941 | *LYZ1* | 2.36 | 2.73E-23 | ENSBTAG00000003751 | *MACC1* | -0.85 | 3.16E-05 |
| ENSBTAG00000000185 | *SLC6A14* | 2.36 | 1.70E-23 | ENSBTAG00000016804 | *LYST* | -0.86 | 6.72E-22 |
| ENSBTAG00000043553 | *GPX3* | 2.34 | 2.40E-60 | ENSBTAG00000017719 | *AKAP6* | -0.86 | 8.18E-07 |
| ENSBTAG00000030913 | *MX1* | 2.28 | 2.43E-25 | ENSBTAG00000009153 | *MLXIPL* | -0.86 | 2.09E-03 |
| ENSBTAG00000039861 | *OAS1Y* | 2.25 | 2.53E-21 | ENSBTAG00000016704 | *SLC37A2* | -0.86 | 1.25E-03 |
| ENSBTAG00000014127 | *PGHS-2* | 2.24 | 1.17E-25 | ENSBTAG00000016223 | *SCRN1* | -0.86 | 1.09E-05 |
| ENSBTAG00000001244 | *PLAT* | 2.22 | 9.76E-56 | ENSBTAG00000004885 | *DDR2* | -0.87 | 3.14E-05 |
| ENSBTAG00000032152 | *TMPRSS6* | 2.17 | 4.89E-20 | ENSBTAG00000009719 | *CALHM2* | -0.87 | 3.49E-06 |
| ENSBTAG00000016061 | *RSAD2* | 2.16 | 4.26E-19 | ENSBTAG00000007447 | *NUDT4* | -0.87 | 1.16E-17 |
| ENSBTAG00000004848 | *OLFML2A* | 2.13 | 8.43E-57 | ENSBTAG00000045699 | *CTNNA3* | -0.87 | 2.00E-03 |
| ENSBTAG00000001060 | *CXCR4* | 2.12 | 2.61E-25 | ENSBTAG00000018165 | *FAM65B* | -0.88 | 1.80E-03 |
| ENSBTAG00000000436 | *TNFAIP3* | 2.11 | 7.35E-48 | ENSBTAG00000014132 | *SNED1* | -0.88 | 1.42E-04 |
| ENSBTAG00000000598 | *CST3* | 2.11 | 3.21E-48 | ENSBTAG00000007429 | *SPNS2* | -0.88 | 3.28E-23 |
| ENSBTAG00000021276 | *TFF3* | 2.10 | 2.56E-19 | ENSBTAG00000002717 | *INA* | -0.89 | 3.04E-07 |
| ENSBTAG00000005615 | *CEACAM1* | 2.09 | 9.02E-20 | ENSBTAG00000010178 | *PTPRD* | -0.91 | 4.56E-04 |
| ENSBTAG00000011316 | *ZC3H12A* | 2.06 | 2.55E-46 | ENSBTAG00000001002 | *TCF7* | -0.91 | 5.67E-14 |
| ENSBTAG00000003313 | *TNFRSF9* | 2.05 | 4.41E-17 | ENSBTAG00000013918 | *LPHN3* | -0.92 | 4.56E-05 |
| ENSBTAG00000017672 | *HAS1* | 2.04 | 3.55E-19 | ENSBTAG00000017456 | *NYAP1* | -0.92 | 9.71E-05 |
| ENSBTAG00000011525 | *SMCT1* | 2.03 | 2.34E-30 | ENSBTAG00000016915 | *FLT1* | -0.94 | 8.05E-07 |
| ENSBTAG00000018280 | *SLC28A3* | 2.02 | 4.27E-20 | ENSBTAG00000009475 | *PLXDC2* | -0.94 | 2.39E-21 |
| ENSBTAG00000007554 | *IFI6* | 1.99 | 1.42E-16 | ENSBTAG00000010498 | *SLC40A1* | -0.95 | 4.68E-08 |
| ENSBTAG00000038154 | *HSH2D* | 1.98 | 5.89E-16 | ENSBTAG00000013537 | *FER1L6* | -0.95 | 1.96E-11 |
| ENSBTAG00000018994 | *TNFSF10* | 1.96 | 2.85E-17 | ENSBTAG00000002880 | *SORBS1* | -0.96 | 1.23E-05 |
| ENSBTAG00000021273 | *SEMA4B* | 1.95 | 2.49E-109 | ENSBTAG00000017549 | *KITLG* | -0.98 | 1.18E-05 |
| ENSBTAG00000015129 | *KLK10* | 1.94 | 2.50E-59 | ENSBTAG00000038093 | *PEG10* | -0.98 | 1.20E-05 |
| ENSBTAG00000014707 | *ISG15* | 1.90 | 1.11E-14 | ENSBTAG00000018706 | *PCTP* | -0.98 | 1.48E-26 |
| ENSBTAG00000045966 | *GPRIN3* | 1.90 | 5.10E-69 | ENSBTAG00000032881 | *SLCO5A1* | -0.99 | 3.13E-05 |
| ENSBTAG00000024406 | *AGR2* | 1.90 | 3.30E-20 | ENSBTAG00000013511 | *VWA5B1* | -0.99 | 1.47E-04 |
| ENSBTAG00000030932 | *IFI44L* | 1.89 | 5.78E-15 | ENSBTAG00000010620 | *ATP6V1B1* | -0.99 | 9.14E-10 |
| ENSBTAG00000002555 | *PDZK1IP1* | 1.87 | 2.13E-14 | ENSBTAG00000007680 | *THSD7A* | -0.99 | 1.90E-04 |
| ENSBTAG00000002898 | *UNC45B* | 1.82 | 5.84E-16 | ENSBTAG00000017722 | *F5* | -1.00 | 9.92E-12 |
| ENSBTAG00000018773 | *RND1* | 1.82 | 4.54E-15 | ENSBTAG00000017709 | *ATRNL1* | -1.00 | 8.39E-05 |
| ENSBTAG00000013452 | *AQP9* | 1.80 | 3.20E-13 | ENSBTAG00000012897 | *ITGA7* | -1.03 | 2.83E-10 |
| ENSBTAG00000030921 | *FAM3B* | 1.80 | 5.58E-36 | ENSBTAG00000026825 | *TMEM37* | -1.03 | 5.81E-17 |
| ENSBTAG00000011487 | *PLEKHS1* | 1.80 | 1.55E-25 | ENSBTAG00000018981 | *TMEM236* | -1.03 | 3.36E-05 |
| ENSBTAG00000045902 | *EPHB2* | 1.79 | 9.60E-16 | ENSBTAG00000006769 | *HSD3B* | -1.05 | 4.00E-14 |
| ENSBTAG00000006505 | *S100A9* | 1.79 | 5.53E-13 | ENSBTAG00000024643 | *VEPH1* | -1.07 | 2.51E-06 |
| ENSBTAG00000012779 | *FAM49A* | 1.79 | 5.79E-40 | ENSBTAG00000012969 | *DKK2* | -1.07 | 6.31E-05 |
| ENSBTAG00000045626 | *DEFB300* | 1.78 | 5.92E-13 | ENSBTAG00000009455 | *IL12RB2* | -1.08 | 5.99E-05 |
| ENSBTAG00000037527 | *OAS1Z* | 1.78 | 1.18E-12 | ENSBTAG00000003568 | *CLDN10* | -1.10 | 4.42E-05 |
| ENSBTAG00000031750 | *PLAC8* | 1.77 | 1.23E-12 | ENSBTAG00000014064 | *FGFR2* | -1.10 | 3.20E-18 |
| ENSBTAG00000017500 | *KCNK12* | 1.74 | 1.78E-13 | ENSBTAG00000008966 | *TSPAN7* | -1.11 | 4.25E-13 |
| ENSBTAG00000006686 | *NPNT* | 1.74 | 1.56E-62 | ENSBTAG00000021830 | *ENPP1* | -1.11 | 6.18E-11 |
| ENSBTAG00000007503 | *STRC* | 1.73 | 3.70E-12 | ENSBTAG00000014046 | *BPI* | -1.11 | 8.11E-10 |
| ENSBTAG00000020674 | *LTB* | 1.73 | 1.42E-17 | ENSBTAG00000012558 | *ADAMTS12* | -1.11 | 8.99E-11 |
| ENSBTAG00000010899 | *TIMP2* | 1.73 | 1.20E-43 | ENSBTAG00000000973 | *KCNJ15* | -1.13 | 7.78E-06 |
| ENSBTAG00000001729 | *DUSP10* | 1.71 | 3.13E-35 | ENSBTAG00000012054 | *ALDH3B2* | -1.15 | 5.81E-17 |
| ENSBTAG00000007423 | *TNFRSF11B* | 1.70 | 6.45E-41 | ENSBTAG00000019554 | *FBP2* | -1.17 | 1.65E-21 |
| ENSBTAG00000017602 | *TMEM45B* | 1.69 | 2.34E-17 | ENSBTAG00000003329 | *FST* | -1.17 | 2.50E-42 |
| ENSBTAG00000047250 | *SPTSSB* | 1.66 | 6.09E-13 | ENSBTAG00000038810 | *SFTA2* | -1.19 | 1.25E-10 |
| ENSBTAG00000019798 | *PIGR* | 1.65 | 7.51E-13 | ENSBTAG00000017369 | *MAMDC2* | -1.19 | 4.99E-10 |
| ENSBTAG00000012519 | *XDH* | 1.64 | 1.28E-66 | ENSBTAG00000047996 | *IFITM10* | -1.21 | 3.21E-07 |
| ENSBTAG00000002288 | *NT5DC4* | 1.63 | 8.12E-11 | ENSBTAG00000009987 | *INTB3* | -1.22 | 3.08E-22 |
| ENSBTAG00000020261 | *CDH26* | 1.63 | 1.07E-15 | ENSBTAG00000020638 | *TIMP3* | -1.24 | 5.13E-33 |
| ENSBTAG00000012406 | *ZBP1* | 1.62 | 1.15E-10 | ENSBTAG00000012994 | *LOX* | -1.25 | 1.02E-09 |
| ENSBTAG00000003196 | *PAPSS2* | 1.62 | 4.21E-16 | ENSBTAG00000033398 | *HKDC1* | -1.26 | 8.12E-21 |
| ENSBTAG00000003152 | *IFI27* | 1.61 | 5.94E-11 | ENSBTAG00000020647 | *RASL11B* | -1.29 | 3.11E-09 |
| ENSBTAG00000034349 | *IFI44* | 1.61 | 1.37E-10 | ENSBTAG00000014189 | *RGS6* | -1.29 | 1.97E-07 |
| ENSBTAG00000012638 | *S100A12* | 1.61 | 1.10E-10 | ENSBTAG00000024560 | *MGC151592* | -1.31 | 8.66E-12 |
| ENSBTAG00000012119 | *PTPRZ1* | 1.60 | 2.10E-10 | ENSBTAG00000014439 | *KMO* | -1.31 | 8.66E-11 |
| ENSBTAG00000030575 | *BHLHE41* | 1.59 | 3.39E-11 | ENSBTAG00000039820 | *CLDN8* | -1.33 | 6.67E-10 |
| ENSBTAG00000014529 | *GBP4* | 1.59 | 1.11E-22 | ENSBTAG00000037795 | *CYP2C87* | -1.33 | 2.63E-07 |
| ENSBTAG00000015094 | *VNN1* | 1.56 | 5.47E-10 | ENSBTAG00000001444 | *TNXB* | -1.33 | 3.68E-09 |
| ENSBTAG00000014921 | *IL6* | 1.55 | 1.65E-10 | ENSBTAG00000038384 | *KRT5* | -1.40 | 9.74E-13 |
| ENSBTAG00000001852 | *BREH1* | 1.55 | 6.26E-10 | ENSBTAG00000026708 | *PRSS35* | -1.57 | 6.81E-24 |
| ENSBTAG00000010408 | *IKBKE* | 1.54 | 4.13E-35 | ENSBTAG00000001652 | *SLCO3A1* | -1.65 | 1.84E-13 |
| ENSBTAG00000016208 | *TGM2* | 1.53 | 2.29E-21 | ENSBTAG00000020194 | *FGF13* | -1.71 | 6.70E-18 |
| ENSBTAG00000005947 | *PLAU* | 1.51 | 3.04E-24 | ENSBTAG00000021118 | *CYP26A1* | -1.76 | 6.50E-13 |
| ENSBTAG00000006523 | *SOD2* | 1.50 | 5.11E-25 | ENSBTAG00000024081 | *ECM2* | -1.81 | 5.67E-15 |
| ENSBTAG00000015086 | *HSD11B1* | 1.49 | 1.58E-25 | ENSBTAG00000006538 | *PTHLH* | -1.92 | 1.32E-18 |
